# Shikimate Kinase-Like 1 Participates in an Ancient and Conserved Role Contributing to Chloroplast Biogenesis in Land Plants

**DOI:** 10.1093/molbev/msaf129

**Published:** 2025-06-02

**Authors:** Michael Kanaris, Jonathan Lee, Belinda Chang, Dinesh Christendat

**Affiliations:** Department of Cell and Systems Biology, University of Toronto, Toronto, ON, Canada M5S 3B2; Department of Cell and Systems Biology, University of Toronto, Toronto, ON, Canada M5S 3B2; Department of Cell and Systems Biology, University of Toronto, Toronto, ON, Canada M5S 3B2; Department of Ecology and Evolutionary Biology, University of Toronto, Toronto, ON, Canada M5S 3B2; Department of Cell and Systems Biology, University of Toronto, Toronto, ON, Canada M5S 3B2

**Keywords:** chloroplast biogenesis, thylakoid network organization, pigment-defective, albino, shikimate pathway, shikimate kinase-like 1, SKL1, protein evolution, *Arabidopsis thaliana*, *Marchantia polymorpha*

## Abstract

Shikimate kinase-like 1 (SKL1) plays an essential role in chloroplast biogenesis in *Arabidopsis thaliana* whereby mutants present a pigment-defective phenotype. The inability to identify SKL1 in organisms predating land plants suggests an important role for this gene coinciding with the emergence of terrestrial plants. A role for SKL1 in chloroplast biogenesis has previously been determined in *Arabidopsis*; however, the biological function for SKL1 has not been established in early land plants. In the present study, we provided functional and evolutionary insights into the diversification of SKL1 in the early land plant *Marchantia polymorpha*. We identified the SK gene homologs common to all land plants, two of which were shown to have high sequence similarity to SK. We confirmed that one member possessed shikimate kinase activity, whereas the second member is inactive. These findings led us to identify *MpSK* (Mp3g21830) and infer the identity of *MpSKL1* (Mp6g03600). Consistent with previous studies in *Arabidopsis*, disruption of *MpSKL1* in *Marchantia* resulted in a pigment-defective phenotype with abnormal chloroplast morphology and thylakoid network organization. Given an early origin of SKL1 in land plant evolution, we investigated requisite structural modifications to an ancestral SK that led to the functional diversification of SKL1. We provided evidence that SKL1 displays an open and accessible substrate binding pocket, conferring its biological function for chloroplast biogenesis. Together, our results demonstrate that the acquisition of SKL1 corresponds with the emergence of terrestrial land plants and that this biological function is conserved across distant plant lineages.

## Introduction

Prevailing theories surrounding the terrestrialization of ancient plants suggest that they were driven by the rapid expansion of molecular processes that contributed to adaptations suited for land. Fossil records and molecular estimates place the emergence of land plants ∼475 to 500 mya (Delwiche and Cooper 2015; Morris et al. 2018), before which aquatic and semiaquatic organisms were the dominant life forms. The physiological need to respond to biotic and abiotic stresses in such environments included adjusting to increased ambient light conditions, limiting tissue dehydration, establishing defense mechanisms, and the ability to grow on terrestrial surfaces (Kenrick and Crane 1997). A transition from water to land occurred within the charophyte freshwater green algae that gave rise to early land plants, with estimates placing the late-diverging Zygnematophyceae as the closest relatives (Domozych et al. 2016). Features among these charophytes shed light on some of the major trends that are associated with prerequisites for the transition to land. For example, a large expansion in some gene families plays important roles in this process, including an increased number of transcription factors and transcriptional regulators for diversified gene regulation, and enzymes involved in polysaccharide biosynthesis for increased cell wall complexity (Nishiyama et al. 2018; Jiao et al. 2020). The presence of a limited number of phenylpropanoids within the flavonoids class in most lineages of green algae was also likely to play pivotal roles in managing increased photooxidative and UV light stress (de Vries et al. 2017).

Liverworts are the earliest identified plants emerging after the transition to land, for which the species *Marchantia polymorpha* was the first genome to be sequenced (Bowman et al. 2017) and has become a popular model organism for studies with an evolutionary focus (Kohchi et al. 2021). Remarkably, biosynthesis pathways leading to the production of specialized metabolites have been identified in liverworts that share features of higher land plant function. For example, liverworts accumulate a wide range of phenylpropanoids and are responsive to abscisic acid during wound, pathogen, and herbivore responses (Yoshikawa et al. 2018; Carella et al. 2019; Romani et al. 2020), photooxidative stress (Kageyama et al. 2015; Soriano et al. 2019; Hernández-García et al. 2021), and desiccation tolerance (Jahan et al. 2019; Godinez-Vidal et al. 2020). The liverwort genome encodes the complete set of components necessary for auxin biosynthesis and signaling pathways derived from the Trp-dependent precursor indole-3-pyruvate (Eklund et al. 2015) and also exhibits auxin-responsive transcriptional gene regulation modulating their growth and development (Kato et al. 2020). The increased genetic diversity of early land plants, facilitated by horizontal gene transfers with symbiotic microbes, fungi (Emiliani et al. 2009; Yue et al. 2012), and the expansion of preexisting gene families in a charophycean algae ancestor (Martin-Arevalillo et al. 2019), required pathways of diverse origins to integrate with one another to provide effective biological responses within a terrestrial environment.

Shikimate kinase-like 1 (SKL1), a gene homolog of shikimate kinase (SK) relating to the shikimate pathway (Herrmann and Weaver 1999), is essential for chloroplast biogenesis in plants. Initial identification and characterization of mutants pertaining to the SKL1 locus were reported in the flowering plant *Arabidopsis thaliana* (Fucile et al. 2008). Two independent T-DNA insertional mutants, *skl1 − 3 and skl1 − 8*, resulted in a pigment-defective phenotype that showed seedling-lethal variegated and albino phenotypes, respectively. SKL1 lacks the ancestral kinase enzymatic activity in vitro, and *skl1*  *−*  *8* mutants accumulate downstream products of the shikimate pathway, suggesting that the pathway is unaffected in this mutant line. Thus, it was demonstrated that SKL1 underwent neofunctionalization and evolved a biological role required for chloroplast biogenesis. Importantly, putative orthologous gene copies of SKL1 were not identified in organisms predating land plants, and therefore, the implications for its role in chloroplast biogenesis relate to a plant-specific process that was likely not established in a charophycean ancestor.

The significance of an expanded repertoire of specialized metabolites and increased genetic diversity supports the land plant transition; however, much less is known about the innovations surrounding chloroplast biology within early land plants. Investigations to date focused on the role of SKL1 have been limited, and its role in chloroplast biogenesis in early land plants remains unknown. In this report, we provide evolutionary insights into the functional acquisition of plant SKL1 focused on the liverwort *M. polymorpha*. We demonstrated that disruption of the *MpSKL1* locus results in a pigment-defective phenotype that is consistent with previous reports in *Arabidopsis*. The encoded gene product, MpSKL1, exhibited modifications through structural divergence in the functional domains associated with the ancestral kinase, leading to neofunctionalization for a biological role that is conserved in plant chloroplast biogenesis. Overall, this study provides evidence for the emergence of SKL1 as a functional protein with a role in chloroplast biogenesis coinciding with the emergence of land plants.

## Results

### Identification of the SK Gene Homologs in the Liverwort *M. polymorpha*

**Fig. 1.**
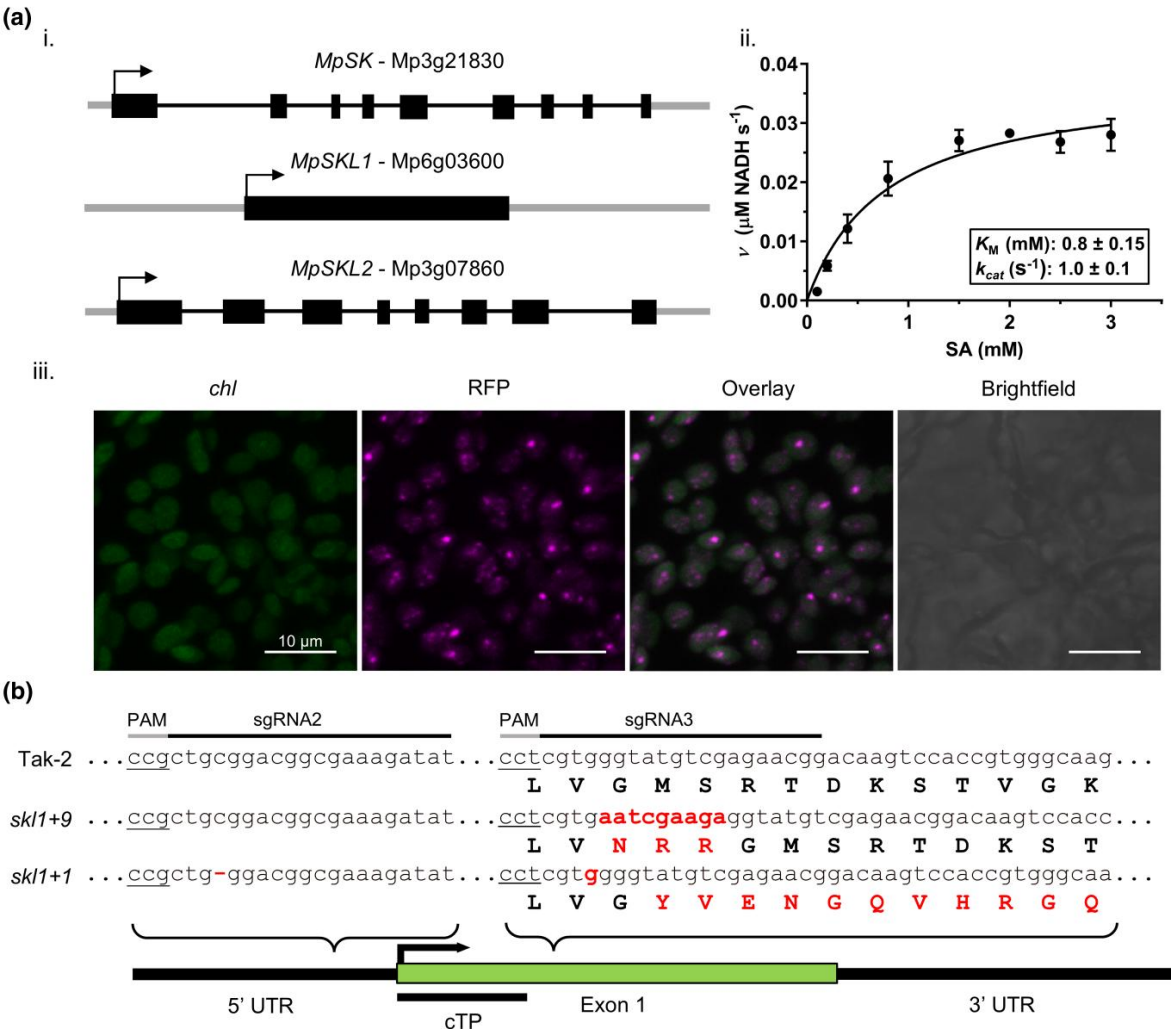
Identification and characterization of the SK family gene homologs in *M. polymorpha.* a) (i) Cartoon representations of the encoding gene structures: exons (rectangles), 5′ and 3′ UTRs (thick lines), introns (thin lines), and start codons (arrows) are indicated. (ii) In vitro Michaelis–Menten saturation curve for Δ113MpSK assayed by a pyruvate kinase–lactate dehydrogenase coupled assay, plotted *ν* (μM NADH s^−1^) versus increasing concentrations of shikimic acid (SA, mM) performed in triplicate with SD measurements where the height of the error bar can be drawn. Kinetic parameters are indicated: *K*_M_ (mM), *k*_cat_ (s^−1^). (iii) Subcellular localization analysis of MpSKL1 C-terminally tagged with RFP. Confocal images were acquired on a Leica TCS SP5 microscope. The full set of images is viewed in supplementary fig. S1, Supplementary Material online. Excitation: 543 nm, emission: tagRFP (578 to 600 nm); chlorophyll (*chl*) (650 to 700 nm). b) Isolation of two independent *MpSKL1* locus mutants generated by CRISPR/Cas9. Locations of two guides targeting the 5′ UTR in relation to the single encoding exon are indicated. Mutations were verified to contain a 9-bp insertion (*skl1*  *+*  *9*), and a single-base-pair insertion (*skl1*  *+*  *1*) in the encoding exon. The resulting inserted or frameshifted amino acids are indicated in the alignment and are further viewed in supplementary fig. S2, Supplementary Material online.

*Marchantia polymorpha* is a popular model plant for evolutionary studies due to its early emergence and having one of the first available sequenced genomes among liverworts. We hypothesized that SKL1 acquired a function in chloroplast biogenesis corresponding with the emergence of terrestrial plants; thus, our studies on *M. polymorpha* served as a basis for investigating this gene in basal land plants. We identified three homologous SK gene sequences in the *M. polymorpha* genome database (MarpolBase) (Bowman et al. 2017; Fig. 1a, i). Two homologs shared high sequence similarity among plant SK sequences (Mp3g21830 and Mp6g03600), whereas the third showed clear separation among SKL2 sequences (Mp3g07860). Notably, both Mp3g21830 and Mp3g07860 have a multiexon gene structure, whereas Mp6g03600 is encoded on a single exon.

In flowering plants, distinctions between SK and SKL1 are made based on previously established consensus sequences in monocots and dicots (Fucile et al. 2008). However, the high degree of similarity and absence of specific sequence features between the proteins encoded by Mp3g21830 and Mp6g03600 confounded the identification of the putative SKL1. Thus, the coding sequences from both genes were cloned and the enzymes subjected to in vitro kinetic analysis by assaying for SK activity. To account for chloroplast transit peptide (cTP) sequence regions, multiple N-terminal truncated constructs were generated for each protein: Mp3g21830 (Δ106, Δ113, Δ115, Δ119) and Mp6g03600 (Δ70, Δ74, Δ76, Δ80), using in silico predictions (Emanuelsson et al. 1999) and previous estimates in *Arabidopsis* (Fucile et al. 2008, 2011). These truncations did not result in any significant differences in recombinant protein expression nor their solubility properties. Enzyme kinetic assays established that Mp3g21830 catalyzed the phosphorylation of shikimate (*K*_M_ 782.5 µM, and *k*_cat_ 1.0 s^−1^) and was consistent with it being a functional SK. The protein encoded by Mp6g03600 was unable to utilize shikimate as a substrate for the various recombinant protein constructs tested in excess of 20 µg per reaction and thus indicated that it did not possess SK activity (Fig. 1a, ii; supplementary fig. S1, Supplementary Material online).

The shikimate pathway is localized within the chloroplast in plants (Mousdale and Coggins 1985; Smart and Amrhein 1987), and while no studies have determined SKL1 localization in planta, a number of high-throughput studies have indicated similar localization in *Arabidopsis* (Ferro et al. 2010; Hooper et al. 2017). We therefore conducted subcellular localization analysis in *M. polymorpha* to verify the accumulation of the full-length protein encoded by Mp6g03600. We generated transgenic *M. polymorpha* plants overexpressing a C-terminal tagged fluorescent RFP reporter for this protein (Fig. 1a, iii; supplementary fig. S1, Supplementary Material online). Consistent with the previous high-throughput studies, this protein colocalized with chlorophyll autofluorescence and thus indicated subcellular localization within the chloroplast. Taken together, these studies led us to establish the function of the gene products Mp3g21830 as *MpSK* and Mp6g03600 as *MpSKL1*.

### Disruption of *MpSKL1* Results in a Pigment-Defective Phenotype

To further investigate the biological role of *MpSKL1*, mutagenesis experiments by CRISPR/Cas9 were carried out in *M. polymorpha*. Expression cassettes targeting the Mp6g03600 locus were designed and transformed into Tak-2 wild type (Fig. 1b). Two independent T1 lines were analyzed, containing either a 9-bp insertion (*skl1*  *+*  *9*), or a single-base-pair insertion (*skl1*  *+*  *1*). The *skl1*  *+*  *1* mutant additionally contained a single-base-pair deletion within the 5′ UTR. The *skl1*  *+*  *9* mutant results in a three-amino-acid insertion in the encoded protein sequence at position 105 (supplementary fig. S2, Supplementary Material online). Due to the single encoding exon within Mp6g03600, the single-base-pair insertion for *skl1*  *+*  *1* results in a frameshift downstream following the predicted N-terminal cTP and produces a truncated protein.

**Fig. 2.**
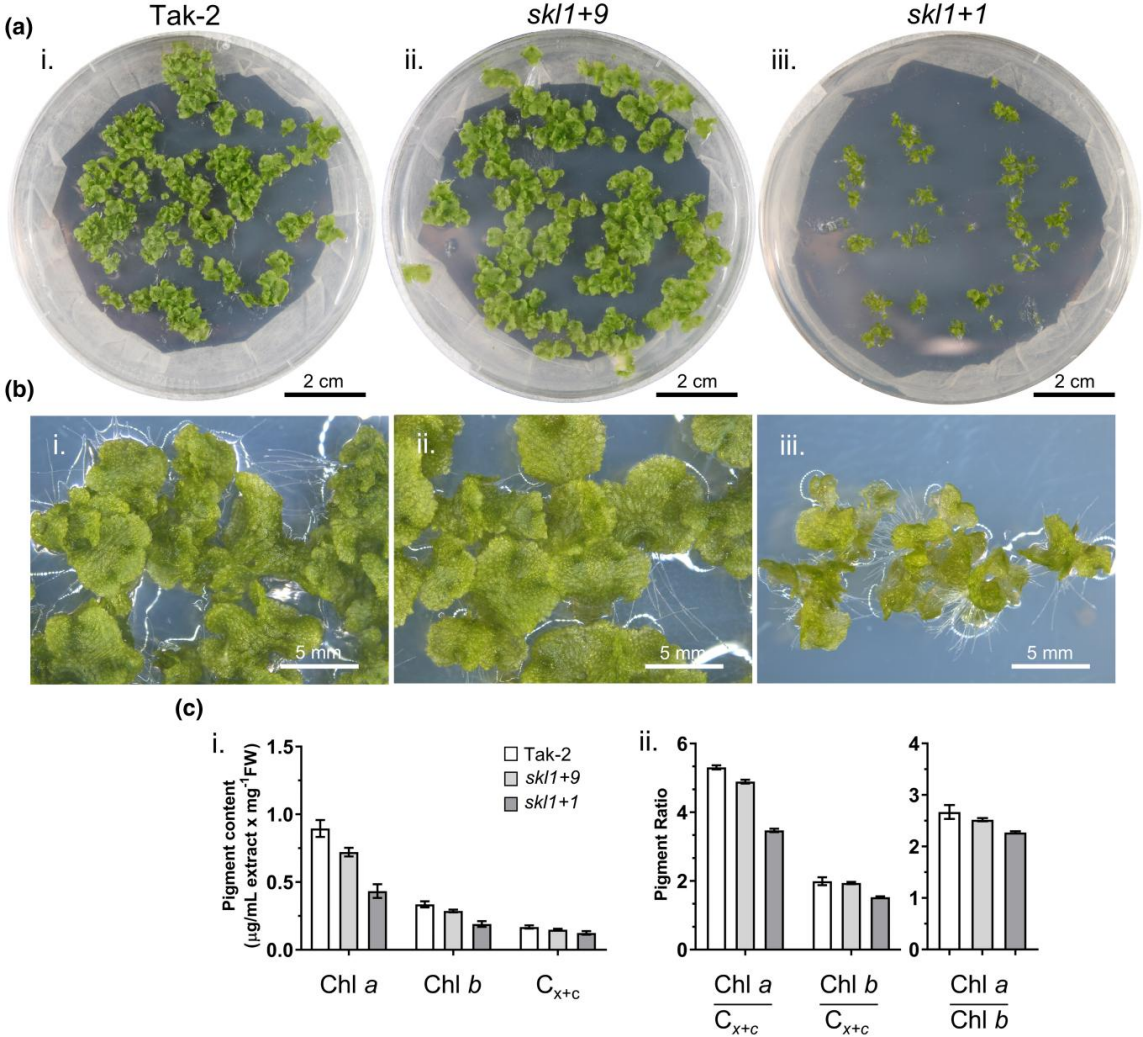
Characterization of pigment-defective *skl1* mutants in *M. polymorpha.* a, b) Photographs of 13-d-old gemmalings from Tak-2 wild-type and the *skl1*  *+*  *9* and *skl1*  *+*  *1* insertion mutants generated by CRISPR/Cas9 mutagenesis. Gemmalings were grown on 0.5× Gamborg plates supplemented with 1.5% sucrose. Zoomed-in photographs of each respective line are indicated in b). Photographs of 28-d-old *skl1*  *+*  *1* gemmalings at a similar developmental stage as the 13-d-old Tak-2 and *skl1*  *+*  *9* are viewed in supplementary fig. S3, Supplementary Material online. c) Quantification of pigment extracts isolated from 11-d-old gemmalings from each respective line. (i) Chlorophyll *a* or *b* and total carotenoids (C_x+c_) levels are indicated, and the respective ratios of each pigment are shown in (ii). Error bars are representative of SD measurements from three biological replicates, where the height of the error bar can be drawn.

Asexually produced gemma in the subsequent generation (G1) was monitored for phenotypic defects from the isolated T1 parent. When grown on media supplemented with sucrose, 0- to 5-d-old gemmalings from *skl1*  *+*  *1* or *skl1*  *+*  *9* did not present phenotypic differences from wild-type plants. Beyond 5 d of growth, *skl1*  *+*  *1* displayed reduced growth and overall pale greening of tissue, and this effect was distinguishable at 13 d (Fig. 2a and b). Extracted total pigments indicated an overall reduction of chlorophylls *a* and *b* by ∼19% and 15% for *skl1*  *+*  *9* and 51% and 43% for *skl1*  *+*  *1*, respectively (Fig. 2c). While the overall chlorophyll content was reduced in these mutants, the ratios of Chl *a*/*b* showed less drastic changes, decreasing by 6% and 15% for *skl1*  *+*  *9* and *skl1*  *+*  *1*, respectively, compared with wild-type plants. Despite the severity of the phenotype, the isolated *skl1*  *+*  *1* mutant was viable and reached an adult stage on media without sucrose, growing approximately at half the rate as wild type and remained pale green (supplementary fig. S3, Supplementary Material online).

**Fig. 3.**
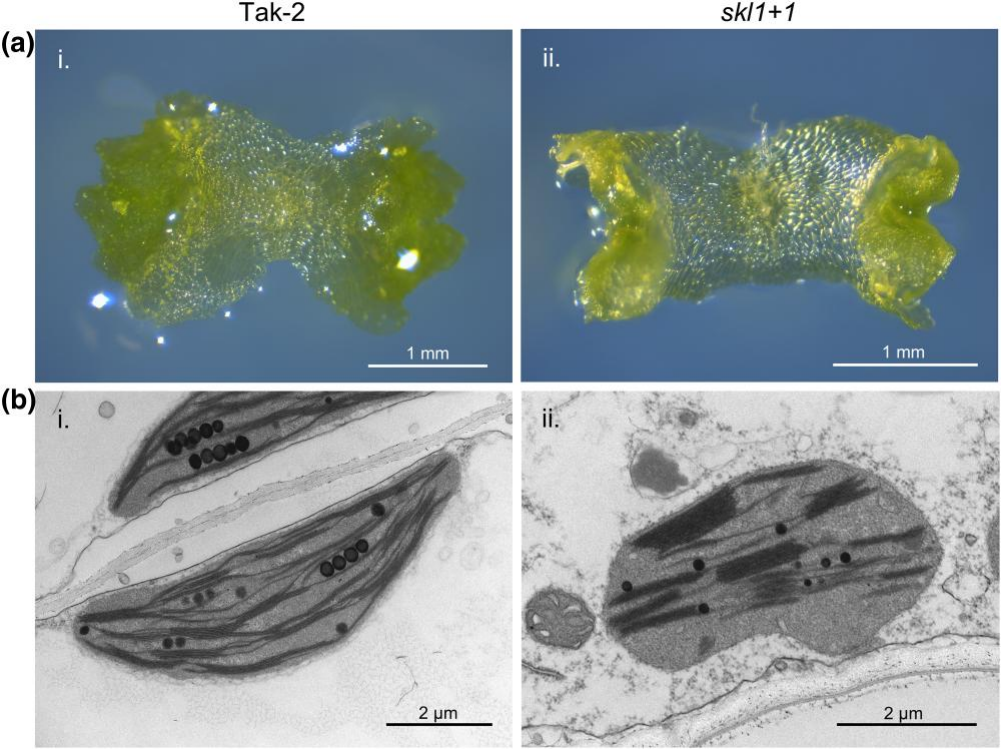
Chloroplast ultrastructure analysis of the *skl1*  *+*  *1* frameshift mutant by transmission electron microscopy. a) Photographs of 10-d old gemmalings from Tak-2 wild type (i) and *skl1*  *+*  *1* (ii) utilized for TEM analysis. Gemmalings were grown on 0.5× Gamborg plates without sucrose supplementation. For clarity, the image shown in (ii) was composited as a stacked photograph derived from two images at separate focal points. b) TEM images of the chloroplast ultrastructure within tissue of Tak-2 (i) and the *skl1*  *+*  *1* mutant (ii). Photographs are representative of the most common plastid type observed along the elongation zones of tissue analyzed. Additional photographs of the observed plastid types are viewed in supplementary fig. S4, Supplementary Material online.

The *skl1*  *+*  *1* phenotype was exacerbated when grown on media without sucrose supplementation. The tissue at the lateral elongation zones experienced the most significant pale-greening effect (Fig. 3a). Thus, downstream analyses were focused on this mutant in the absence of sucrose to detail the *MpSKL1* phenotype. Our results were consistent with impairment of chloroplast biogenesis, and thus, the chloroplast ultrastructure of gemmalings was analyzed by transmission electron microscopy. Representative images from Tak-2 plants showed consistent crescent-shaped structures with elongated thylakoid membranes and grana stacking among the chloroplasts observed (Fig. 3b, i). In comparison, the *skl1*  *+*  *1* mutant displayed impaired and highly variable chloroplast morphology. The most consistent chloroplast type observed is represented in Fig. 3b, ii containing smaller, more rounded structures with less prevalent thylakoid membrane networks. Developmental impairment for the observed chloroplasts varied from cell to cell (supplementary fig. S4, Supplementary Material online).

Semiquantitative expression analysis indicated that *skl1*  *+*  *1* gemmalings accumulated higher levels of SKL1 transcript compared with wild type (supplementary fig. S5, Supplementary Material online). Sequencing of the resulting *SKL1* cDNA from this mutant line showed a predominant chromatogram profile that was obtained from a frameshifted transcript attributed to the *skl1*  *+*  *1* insertion. It is important to note that within the chromatogram profile for this mutant, a signal of low-intensity peaks was observed that corresponded to the presence of a small proportion of wild-type transcript being produced together with this mutant transcript. To further investigate this finding, we monitored the *skl1*  *+*  *1* mutant line in subsequent generations (G2) and identified a segregating population of gemmalings (supplementary fig. S5b, Supplementary Material online). We observed a low frequency of plants that were darker green in appearance in comparison with predominantly pale green plants. Despite our efforts, we were unable to attain a homogenous mutant population based on cDNA sequencing of individual plants.

### MpSKL1 Functionally Complements the Loss of AtSKL1 in Chloroplast Biogenesis

The appearance of SKL1 corresponding with the emergence of ancient land plants led us to hypothesize that SKL1 participates in a highly conserved process related to chloroplast biogenesis. Given this role, we focused our investigation on the functional conservation of SKL1 across distant plant lineages. We independently expressed both MpSKL1 and MpSK in the *Arabidopsis skl1*  *−*  *8* mutant background and focused on their ability to rescue chloroplast biogenesis as a read-out for functional complementation. To ensure correct localization of the ectopic constructs within *Arabidopsis*, we fused the predicted cTP from AtSKL1 to MpSKL1 or MpSK lacking their own endogenous transit peptide (AtcTP-Δ80MpSKL1 or AtcTP-Δ113MpSK). These constructs were generated with a C-terminal mGFP5 fluorescent reporter under a 35S promoter (Earley et al. 2006).

**Fig. 4.**
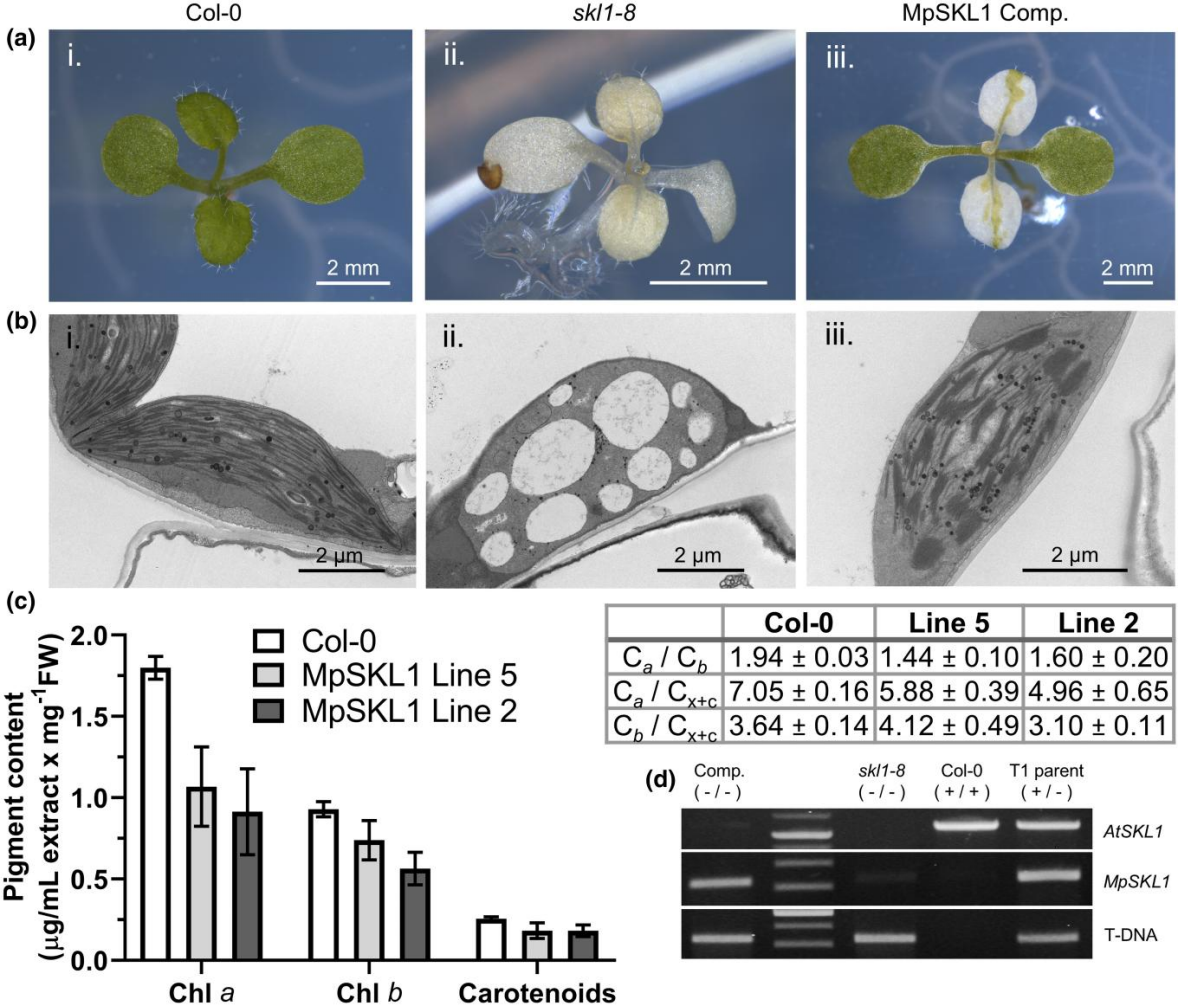
Rescue of chloroplast biogenesis by MpSKL1 in the *Arabidopsis skl1*  *−*  *8* mutant. a) Photographs of transgenic *A. thaliana* T2 generation seedlings overexpressing MpSKL1 in a homozygous *skl1*  *−*  *8* mutant background (iii, 12 d old) in comparison with Col-0 wild type (i, 10 d old) and the homozygous *skl1*  *−*  *8* T-DNA insertional mutant (ii, 10 d old). b) Transmission electron microscopy of the chloroplast ultrastructure observed within the cotyledons of Col-0 (i), *skl1*  *−*  *8* (ii), and MpSKL1 complement line (iii). Additional high-magnification images of the MpSKL1 complement line are viewed in supplementary fig. S6, Supplementary Material online, and images of the true leaf plastids are viewed in supplementary fig. S7, Supplementary Material online. c) Quantification of pigment content and calculation of the respective pigment ratios isolated from whole seedlings of Col-0 (10 d) compared with two independent MpSKL1 complementation lines (12 d). Chlorophylls *a*, *b* (Chl *a*, *b*); carotenoids (C_x+c_). d) Verification of loci by genotyping analysis of the *skl1*  *−*  *8* T-DNA insertion, endogenous *A. thaliana SKL1* gene, and the transgenic *MpSKL1* construct. Individual seedlings depicted in a) (Comp.) were subjected to genotyping in addition to a transgenic heterozygous *skl1*  *−*  *8* control sample (T1 parent).

Transgenic lines were segregated from a heterozygous *skl1*  *−*  *8* plant and monitored for complementation of the homozygous *skl1*  *−*  *8* seedlings. Progeny from the transgenic seedlings overexpressing AtcTP-Δ80MpSKL1 rescued chloroplast biogenesis within the cotyledons of the *Arabidopsis* seedlings (Fig. 4a). Diffuse pale green and yellow tissue was noted along the developing central leaf vein extending to the tips of the leaf. Transmission electron microscopy was carried out to assess the ultrastructure of chloroplasts within these sectors. Representative chloroplasts observed from the rescued cotyledons are depicted in comparison with the Col-0 and homozygous *skl1*  *−*  *8* albino controls (Fig. 4b). The chloroplasts were morphologically and structurally comparable to wild type; however, they contained a much higher degree of thylakoid membrane stacking. The increased density of appressed grana was identified in the majority of chloroplasts, of which some were exaggerated (supplementary fig. S6, Supplementary Material online), whereas this phenotype was not seen in any of the wild-type sections under our growth conditions. Pale green or yellow sectors along the true leaf central vein in the MpSKL1 complemented line were further analyzed. These plastid types predominantly resembled that of *skl1*  *−*  *8* vesiculated structures; however, we occasionally observed partially developed chloroplasts that appeared with circular morphology and low levels of thylakoid membranes (supplementary fig. S7, Supplementary Material online).

The overall chlorophyll production in these partially rescued seedlings showed restoration to ∼55% and 70% of wild-type levels for chlorophylls *a* and *b*, respectively (Fig. 4c). The genotype of individual seedlings confirmed that these phenotypes were observed in a homozygous *skl1*  *−*  *8* insertion background ectopically expressing *MpSKL1* (Fig. 4d). Despite the appearance of rescued chloroplast biogenesis in the cotyledons, this was not sufficient for proper growth and development of these seedlings past 1.5 to 2 weeks of age. Tissue greening was restricted to the cotyledons and was not apparent in the true leaves. To further investigate the localization of this ectopic construct, green tissue sectors were analyzed by confocal microscopy in which we observed colocalization of the GFP signal with chlorophyll autofluorescence (supplementary fig. S8a, Supplementary Material online). Analysis of transgenic plants expressing AtcTP-Δ113MpSK did not show functional complementation of the *skl1*  *−*  *8* albino seedlings in the lines analyzed (supplementary fig. S8b, Supplementary Material online).

### Alterations in the LID and Shikimate Pocket Resulted in MpSKL1 Functional Divergence

The neofunctionalization of an ancestral SK likely played a primary role in the evolution of SKL1 function. The rescue of chloroplast biogenesis in the cotyledons of *skl1*  *−*  *8* seedlings by MpSKL1 and not by MpSK further supports this model. We focused our studies on these two highly similar gene homologs to explore the evolutionary trajectory of SKL1, with the goal of identifying sequence signature motifs that are being modified to confer SKL1 biological activity. We posit that these signatures should be observed in the 3D structure of the protein, especially if they deviate from the ancestral kinase and if they occur within the microenvironment of the ligand binding region.

**Fig. 5.**
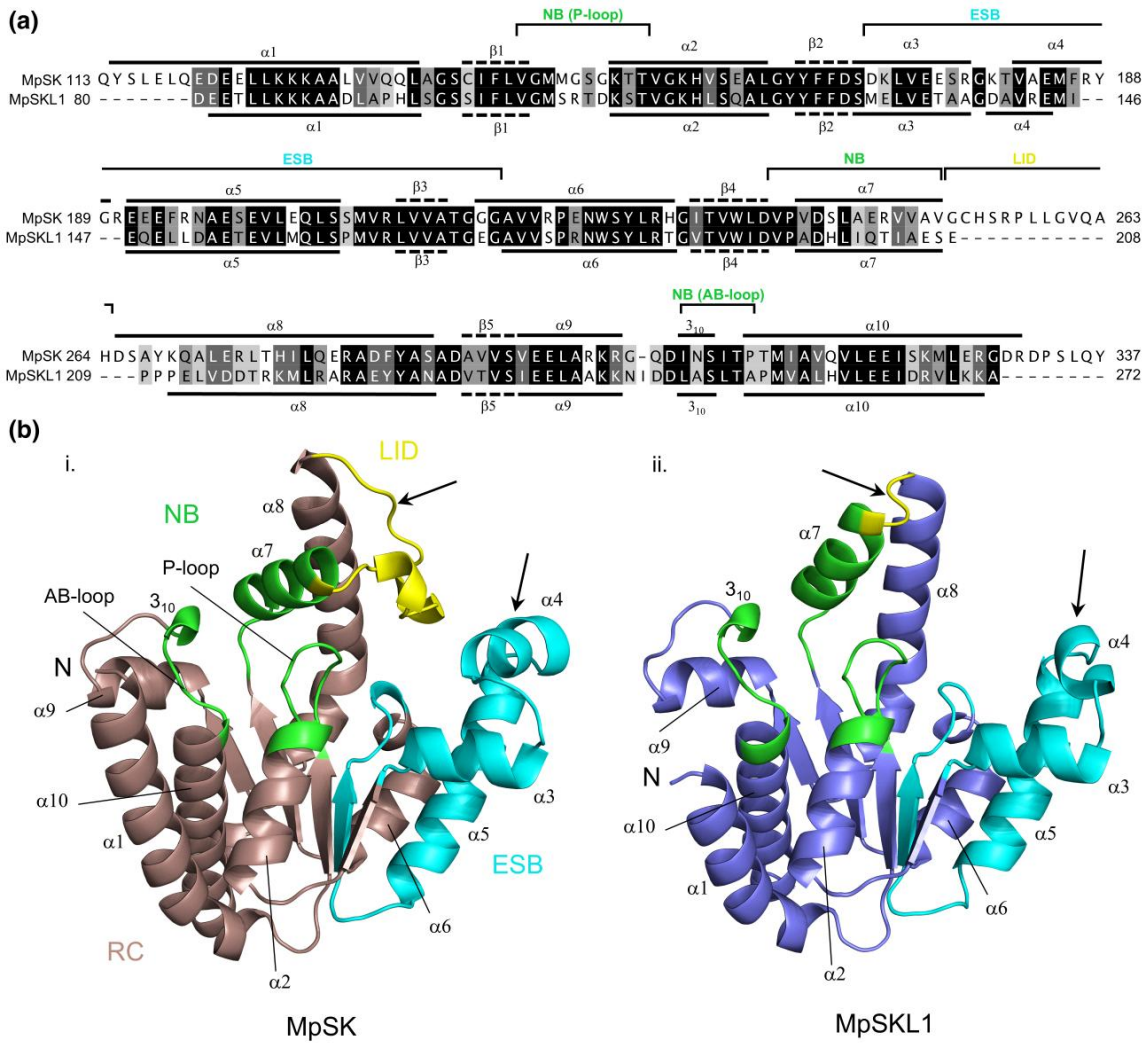
Comparative structural analysis of MpSK and MpSKL1 by predictive modeling. a) Amino acid sequence alignment of Δ113MpSK with Δ80MpSKL1, highlighting secondary structure predictions: α-helix (solid line) and β-sheet (dashed line). Sequence alignment was generated by ClustalOmega1.2.4 and annotated using JalView. b) Structural models for MpSK (i) and MpSKL1 (ii). SK domains corresponding to the reduced core (RC), ESB, nucleotide binding domain (NB), and LID with the corresponding homologous regions for MpSKL1 are indicated. Arrows indicate regions of most significant divergence between the two models. Structural models were generated using the Robetta RoseTTAFold prediction service and were visualized in PyMOL 2.5.

We modeled and compared the structures of MpSK and MpSKL1 using the Robetta protein structure prediction server (Kim et al. 2004). Amino acid sequences without the cTPs were analyzed by the RoseTTAFold prediction server (Baek et al. 2021). The theoretical models for both MpSK and MpSKL1 adopted similar 3D folds (Fig. 5). The accuracy of these theoretical models awaits the determination of their structure; however, both overall structures are highly similar to the crystal structure of the bacterial *Mycobacterium tuberculosis* SK (MtSK) (Hartmann et al. 2006) and plant *Arabidopsis* SK2 (AtSK2) (Fucile et al. 2011) enzymes, thus supporting downstream analyses. Specifically, this included the regions corresponding to the nucleotide binding (NB) domain, and a significant portion of the extended shikimate binding (ESB) domain (Gu et al. 2002; Gan et al. 2006; Hartmann et al. 2006). The major differences between the two models lie within the substrate binding pocket (Fig. 5b, arrows). With respect to MpSK, the shikimate pocket includes a LID domain and a short helix, α4, both of which are close to each other and serve to physically cap the substrate binding site. This LID domain is absent in MpSKL1, and the corresponding α4 helix is shortened to a single turn in the structure. These alterations in MpSKL1 result in a substrate binding pocket which is freely accessible to the bulk solvent.

The largest contributions to sequence divergence between the two proteins are contained within the LID, identified as the loop joining α7 to α8, and α4 in the ESB domain. These two structural differences are consistent with the sequence gaps observed in the alignment between MpSK and MpSKL1. In contrast, the NB domain comprising the adenosine binding loop (AB-loop) and phosphate loop (P-loop) adopts a nearly identical conformation between the two proteins. Exception to this observation is the positioning of α7 for MpSK which is nearly perpendicular to α8, where in MpSKL1 is almost parallel.

### Electrostatic Surface and Substrate Binding Domain Remodeling of MpSKL1

**Fig. 6.**
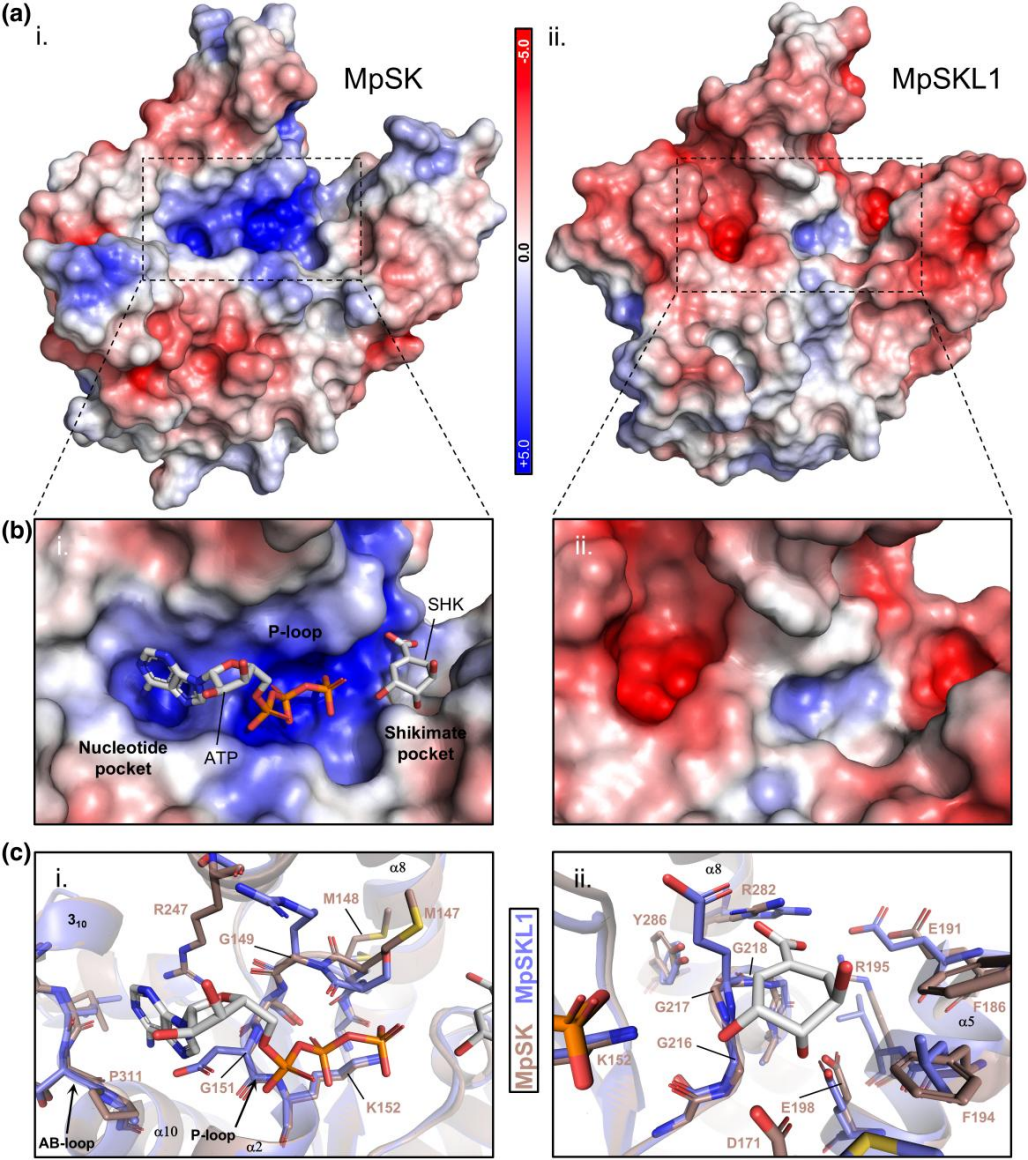
Surface electrostatic charge and binding pocket comparisons of MpSK and MpSKL1. a, b) Electrostatic charge visualizations were calculated using the APBS extension in PyMOL 2.5 and were projected onto the surface of MpSK (i) and MpSKL1 (ii) structures. For clarity, MpSK was modeled without its LID domain to compare the structures in an open conformation. To visualize the nucleotide binding pocket of MpSK, docking analysis with ATP was performed using AutoDock Vina. The substrate binding pocket was visualized by overlaying the MtSK crystal structure solved with bound shikimate. c) Residues contributing to the nucleotide (i) and substrate (ii) binding pockets of MpSK were overlaid with the corresponding region within MpSKL1 for comparison of the modified pocket. Residues are labeled with respect to the full-length proteins and were visualized in PyMOL 2.5.

Characteristic of MtSK crystal structures is the formation of a positively charged electrostatic surface in the NB and ESB pockets required for ATP and shikimate binding (Hartmann et al. 2006; supplementary fig. S9a, Supplementary Material online). Moreover, an exposed channel connecting these two pockets comprising the P-loop similarly exhibits a positive electrostatic potential. This channel serves to bind the triphosphate and bring the λ-phosphate in close proximity to shikimate for phosphoryl transfer, and this process is assisted by an arginine in the LID domain having the consensus RPLL motif. These structural features are all retained in the AtSK2 crystal structure (PDB ID: 3nwj), which suggests that the conservation of this positive surface potential in the plant enzyme is also important for the binding of both ligands (supplementary fig. S9b, Supplementary Material online). Similar characteristics were also seen for the modeled MpSK structure (Fig. 6). In contrast, MpSKL1 exhibited changes for its surface charge that indicated a shift toward a negative electrostatic potential in these regions. Both corresponding pockets in MpSK involved in nucleotide binding and substrate binding showed negative electrostatic surface potentials in MpSKL1. The region corresponding to the P-loop, in contrast, partially retained a positive electrostatic surface potential.

Analysis of the ESB domain of MpSKL1 identified specific amino acid residues that may be associated with the evolution of substrate preference for SKL1. Analyses were conducted by comparison to the shikimate bound MtSK structure. The second glycine in the GGG loop of MtSK hydrogen bonds with the 3′ hydroxyl of shikimate (G217 in MpSK), and a glutamate residue was substituted at this position in MpSKL1, which results in steric interference with the carboxyl group of shikimate. Within the Walker B DSD motif, the second aspartate provides interactions to coordinate the 3′ and 4′ hydroxyl groups of shikimate (D171 in MpSK), and a methionine is found at this position in MpSKL1. Overall, most residues that contribute to the architecture of the substrate binding pocket are conserved between MpSK and MpSKL1 (E191, E198, G216, G218, R282, Y286 in MpSK). The most significant changes are made to two bulky phenylalanine residues (F186 and F194 in MpSK) and as a result opens the modified substrate pocket in direction of the shortened α4-helix in MpSKL1.

We further identified substitutions within the NB domain contributing to the partial remodeling of the nucleotide binding pocket in MpSKL1. With relation to MpSK at residues R247 from α7 and P311 of the AB-loop, both of which form close contacts with the nucleoside of ATP in the microbial SK structures, substitutions to T203 and A254 are observed at these positions, respectively. Two other substitutions lie within the P-loop of MpSK, G149 and G151. In MpSKL1, an arginine and aspartate are substituted at these positions, respectively, which reveal an enlarged, electronegative pocket.

### Extensive Modifications of the LID and ESB Domains Drove SKL1 Diversification Within the Plant Kingdom

Structural determinants indicated that the evolution of the biological function for MpSKL1 involved modifications of the LID and ESB domains. These observations have been made in *M. polymorpha*; however, sequencing efforts in recent years have enabled the analysis of a large number of plant sequences. We therefore carried out a comparative analysis of SK and SKL1 sequenced transcriptomes from the OneKP database to explore their diversification within the plant kingdom (Leebens-Mack et al. 2019). We focused on the specific protein sequence signatures that are associated with SK and SKL1 function based on our structural modeling of MpSK and MpSKL1.

**Fig. 7.**
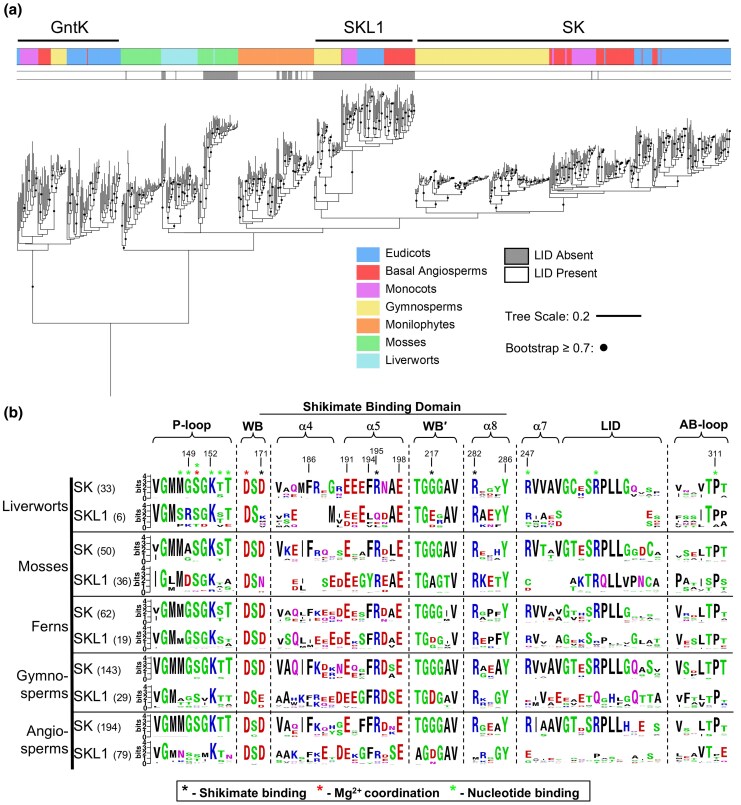
Phylogenetic reconstruction and sequence analysis of SK and SKL1 gene homologs distributed within the plant kingdom. a) The 1,000 Plants (OneKP) project was queried for the SK and SKL1 gene homologs. Phylogenetic analysis was conducted by the neighbor-joining method using a Poisson model of amino acid substitution with 1,000 bootstrap replicates. Plant lineage identifications are indicated according to classifications by the OneKP project. Sequences were analyzed for deviations from the SK LID RPLL consensus motif (gray bars). Multiple sequence alignments were carried out using ClustalOmega and the final tree was displayed using iTOL, with further annotations using Inkscape. Nodes supported by ≥70% bootstrap scores are indicated with black circles and the final tree was rooted on the glucokinases outgroup. b) WebLogo consensus sequences for functional regions contained within the nucleotide and shikimate binding domains. Residue numbers are according to the positions within the MpSK structure as reference, and those that participate in ligand, substrate, or metal cofactor interactions within the MtSK crystal structures are indicated (*). WB, Walker-B; WB′, Walker-B second chain.

Identification of SKL1 sequences distributed within the plant kingdom was made based on deviations within the conserved RPLL motif of the SK LID domain (Fig. 7a). We performed structural modeling for representative species within each lineage (supplementary figs. S10 and S11, Supplementary Material online) and detailed sequence changes by comparison to the respective SK gene homologs (supplementary figs. S12 to S16, Supplementary Material online). Overall, trajectories for SKL1 within the plant kingdom were marked by significant sequence and structural diversity. Similar to MpSKL1, SKL1 sequence divergence from SK is primarily associated with changes to the LID; however, most orthologs exhibit lineage-specific trends within this region. For example, liverworts and mosses include large truncations in this domain by comparison to their respective SK gene homologs, whereas monilophytes (ferns) and gymnosperms (conifers) show similarity in the length of the LID domain but contain significant sequence variation. Angiosperms including eudicots, monocots, and other early-diverging species are marked by both of these features. Based on these observations, we hypothesized that persistent and adaptive mutations may have played a role in the evolution of this gene homolog during the widespread expansion of plants. We analyzed DNA coding sequences with PAML to estimate the rates of positive selection for SKL1 (Yang 2007). However, using codon-specific measures for selective pressure, we did not find significant evidence for positive selection driving the evolution of SKL1 within the plant kingdom (supplementary tables S4 to S6, Supplementary Material online).

Equivalent residues from the microbial MtSK crystal structures that participate in shikimate interaction are R282, R195, G217, and D171 in MpSK (Fig. 7b). G217, found at the N-terminus of α6 within the GGG loop, is mutated among almost all SKL1 orthologs to either an aspartate or glutamate. All SKL1 sequences excluding angiosperms retain R282 within α8, and less common was the loss of the Walker B D171 which was identified only in the bryophyte lineage including liverworts and mosses. Other residues that contribute to the formation of the substrate pocket stemming from α5 and α8 are largely conserved between SK and SKL1 sequences. While various substitutions are found within the P-loop compared with SK, the modeled structures for these plant lineages retain the disordered loop characteristic of this motif, which also accommodates enough space for the triphosphates of an ATP ligand.

## Discussion

### The Emergence of Liverworts and the Earliest Functional Diversification of SKL1

This study investigates the evolutionary diversification and conservation of function of the SKL1 gene homolog. Consistent with observations for higher land plants, the identification of SKL1 in *Marchantia* places its emergence to coincide with the terrestrialization of land plants. In this analysis, we determined that MpSKL1 is a neofunctionalized gene derived from an ancestral SK. As indicated by the gene structure for *MpSKL1*, this was likely mediated by a retrotransposition insertion event, resulting in the duplication and genomic reintegration of the coding sequence of the parental gene (Panchy et al. 2016). Recombinant in vitro enzyme activity assays established the biochemical functions for each highly similar gene homolog in *Marchantia*. Given an origin for SKL1 in liverworts, the overlap in sequence signature to SK is likely related to the limited timeline for the functional diversification of the ancestral gene duplicate. This is especially apparent by comparison of SK and SKL1 sequences among distantly related seed-bearing plants, including gymnosperms and angiosperms, as these sequences show clear delineation in sequence motifs and phylogenetic clustering for each respective gene homolog.

Features for the evolution of novel protein function derived from gene duplication events often involve selection within specific functional domains of the descendant gene, thus allowing it to fulfill its necessary role to become neofunctionalized (Clifton et al. 2018; Bradley and Beltrao 2019). Our bioinformatics analysis provided insights into the changes for these functional domains associated with the SKL1 gene homolog, starting from the early copy in liverworts leading to some of the more recently evolved plants. The diversity of the SKL1 orthologs indicated variable patterns of sequence divergence that were specific to major lineages of plants. This was especially apparent in the LID domain, as well as for sites within the P-loop and Walker B motif. Given this sequence diversity, we initially hypothesized that SKL1 evolution was associated with persistent and recurring mutations within these functional domains of this gene homolog. However, we did not observe sufficient evidence that positive selection by codon-based substitution patterns played a major role in the evolution of SKL1 function. We speculate that this is the result of factors relating to the overall conserved function of both the SK and SKL1 gene homologs. Firstly, the findings from our study support a model by which SKL1 acquired an essential biological function for chloroplast biogenesis early in plant evolution. It is therefore likely that the potential for this gene to acquire adaptive mutations was restricted during widespread plant diversification. This notion is consistent with our reciprocal ectopic expression analysis, as the biological role for SKL1 in *Marchantia* is similar to that in *Arabidopsis* and indicates a conserved process. Furthermore, limited evidence of positive selection may also relate to the essential role of the parental SK gene copy. The evolution of SKL1 function involved repurposing of a conserved kinase involved in the shikimate pathway. These changes were therefore constrained to a limited number of sites within the protein, whereas most other structural elements remained unchanged to preserve the overall structure of the ancestral kinase. Mutations within an ancestral kinase leading to its neofunctionalization toward SKL1 were likely acquired early in the evolution of these plant lineages and quickly became fixed under purifying modes of selection. Lastly, we also consider that detection of codon-specific signs of positive selection using the various evolutionary models may have also been impeded by the poor alignments within some sequence regions of interest, especially the LID domain, that show a high degree of diversity among the SKL1 orthologs and thus may lead to difficulties for estimations of positive modes of selection.

Analysis of the two mutant *skl1* lines produced notable results that require further discussion. For *skl1*  *+*  *1*, we were unable to isolate a homogenous population for the frameshifted transcript. Based on our sequencing analysis, we found that the subsequent generations of *skl1*  *+*  *1* progeny always contained low levels of wild-type transcript. This is attributed to a scenario in which this line is a chimeric mixture of different cell types. In support of these observations, chimeric phenotypes have been reported in *M. polymorpha* using similar methods (Sugano et al. 2018). Parallels can be drawn to the *skl1*  *−*  *3* phenotype in *Arabidopsis* whereby we observed similar pale green phenotypes for the isolated *skl1*  *+*  *1* mutant. The *skl1*  *−*  *3* phenotype might be attributed to perturbations of mRNA splicing due to the T-DNA insertion within the third intron. Similarly, the low abundance of wild-type transcript for the *skl1*  *+*  *1* mutant line is likely correlated with highly reduced levels of functional protein in these plants. To the same extent observed for *skl1* mutants in *Arabidopsis* seedlings, it is expected that knockout of *MpSKL1* is lethal and thus isolation of these plants would require alternative conditional knockout methods to probe gene function (Nishihama et al. 2016; Sugano et al. 2018).

### SKL1 Functional Divergence Involves a Modified Substrate Pocket that is Accessible to the Bulk Solvent

We investigated the structure of MpSKL1 to detail specific changes that led to an acquisition of its novel biological function in chloroplast biogenesis. A combination of in planta studies supported by sequence analysis and 3D structural modeling provided a unique opportunity to explore the neofunctionalization of SKL1 as a model for functional innovation. Comparative analysis of the theoretical models to MtSK and AtSK2 crystal structures provides evidence that MpSKL1 biological function is associated with the loss of the LID domain and a portion of the ESB derived from the ancestral SK gene duplicate. The evolution of SKL1 function derived from modifications of structural elements within the ancestral SK binding site is consistent with the notion that selection pressures driving protein neofunctionalization are often dictated by remodeling of established functional sites (Clifton et al. 2018; Bradley and Beltrao 2019). Therefore, it is expected that this structural reorganization resulted in the evolution of a new ligand binding site conferring SKL1 function. The modification of these two domains produces a more open and accessible substrate binding site, and although the identity of a ligand has not been made, the overall architecture and physical dimensions of this portion of the protein strongly indicate that it is sufficient to accommodate a small molecule ligand. The shift toward a negative electrostatic surface potential in the modified binding site for MpSKL1 is expected to be important for binding through electrostatic complementarity, for which the ligand itself may possess positively charged functional groups (Cons et al. 2022). The reengineered active site is found to be constrained within the substrate binding site of the protein that is closely associated with the P-loop and thus may still allow for phosphoryl transfer from an ATP molecule.

### *skl1* Phenotypes are Associated With Perturbations in Thylakoid Network Organization

The ultrastructure of the chloroplasts rescued by MpSKL1 within the cotyledons of *Arabidopsis* seedlings indicated unusual organizations associated with their thylakoid networks. Grana height within the appressed regions appeared much taller compared with wild type, suggesting that perturbations in the organization of these membranes were caused by the ectopic expression of *MpSKL1*. Extensive studies have detailed the highly dynamic nature of thylakoid networks in relation to state transitions (Longoni and Goldschmidt-Clermont 2021). Regulation of the phosphorylation status of LHCII trimers (e.g. LHCB 1/2) and photosystem core subunits (e.g. Psb A/D/H) is mediated by the STN7 and STN8 kinases (Bellafiore et al. 2005; Bonardi et al. 2005) and also involves the antagonistic effects by the PPH1 (TAP38) (Shapiguzov et al. 2010) and PBCP phosphatases (Bellafiore et al. 2005; Bonardi et al. 2005). Isolated mutants for genes involved in state transitions have been reported to have drastic effects on thylakoid network organization (Fristedt et al. 2009; Samol et al. 2012; Armbruster et al. 2013; Ancín et al. 2019). The connection between state transitions and thylakoid network reorganizations is hypothesized, at least in-part, to be coordinated by the activation of key reorganization factors by the STN7/8 kinases. This has been suggested based on the STN8-dependent phosphorylation of CURT1b (Shapiguzov et al. 2010), a member of a well-characterized family of proteins influencing thylakoid architecture. This gene family also displays atypical grana stacking in overexpression lines (Pribil et al. 2018; Sandoval-Ibáñez et al. 2021) and higher-order mutants (Armbruster et al. 2013). Extensive thylakoid networks and tall grana stacks within the chloroplasts rescued by MpSKL1 having lowered Chl *a/b* ratios are consistent with these studies. Further, the progressive decline in the Chl *a/b* ratio in both *skl1* mutants in *Marchantia* suggests a related response, which also appears to be correlated with the severity of the pale green phenotype. The ability for chloroplasts to assemble and disassemble grana stacks is a plant-specific adaptation which is necessary to adequately adjust to fluctuating lighting conditions in various environments (Nevo et al. 2012). While there have been some gains in ultrastructural complexity in the thylakoid networks of charophycean algae including Zygnematophyceae (Stamenković et al. 2014), an origin for these processes remains poorly understood but was likely adapted during the transition to land (Ostermeier et al. 2024; Perez-Boerema et al. 2024). The phenotypes for MpSKL1 and similarity to processes affecting thylakoid network organization offer possible insights into a more specific role for SKL1 coinciding with the transition to land and will thus require future investigations.

## Concluding Remarks

**Fig. 8.**
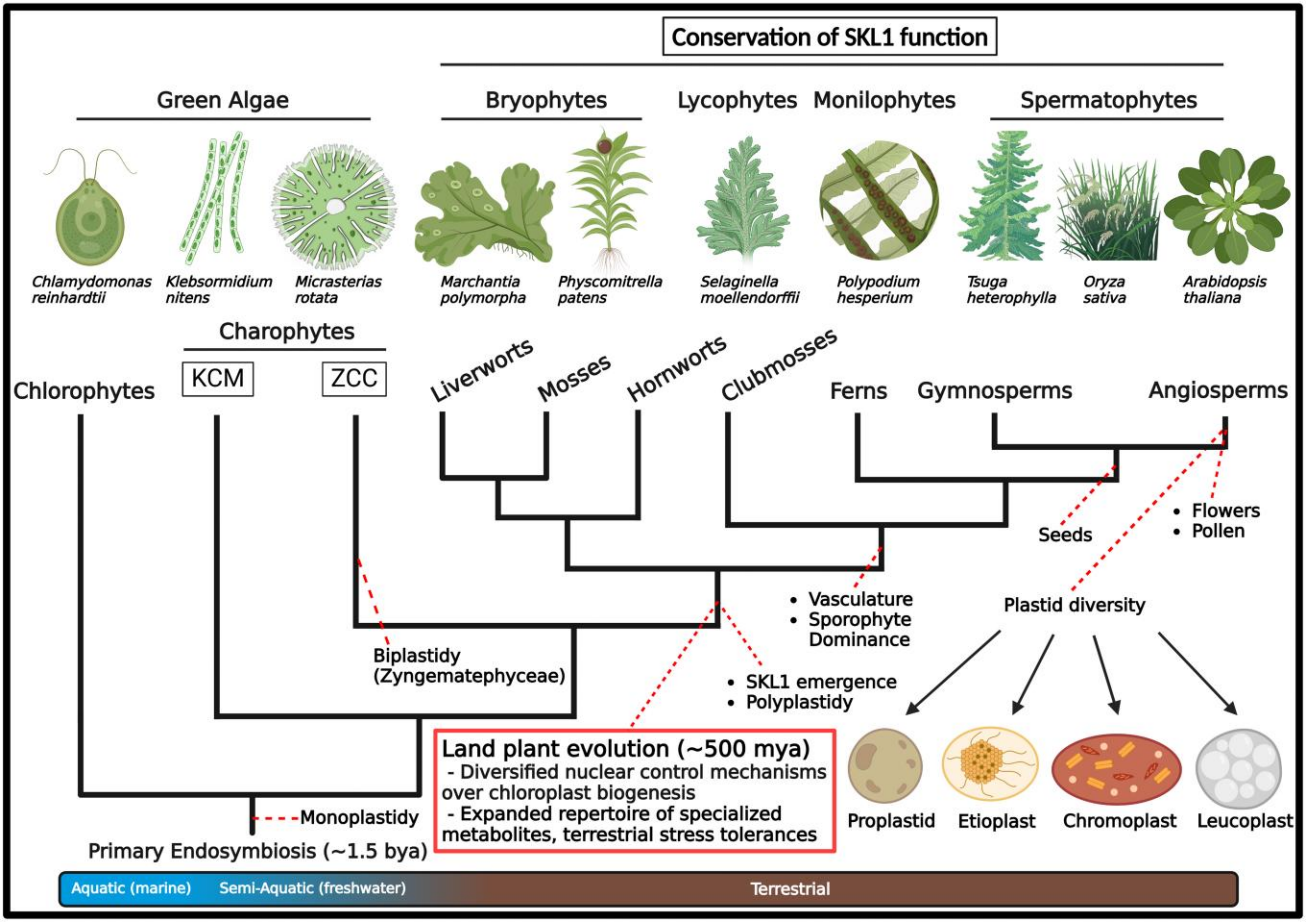
The emergence of SKL1 in early plants coincides with the transition to land. Terrestrialization by early plants required adaptations to molecular processes to accommodate stresses on land. This was facilitated by increased genetic diversity and an expanded repertoire of specialized metabolites. Significant among these adaptations were the adoption of plant cells housing multiple chloroplasts, derived from a single or biplastid-containing charophycean algae ancestor of the late-diverging Zygnematophyceae. In this manner, SKL1 played a pivotal role for plant nuclear genomes to regulate the biogenesis of chloroplasts in a highly coordinated manner. Liverworts are the earliest identified land plants encoding SKL1 with a biological function that is conserved across distantly related relatives, including those of flowering plants such as *Arabidopsis*. The divergence of SKL1 sequence and structure within higher land plants may reflect adaptations for increased complexity of regulation for SKL1 function, such as a role in the biogenesis of specialized plastids found in higher land plants. Figure created with BioRender, https://www.BioRender.com/h0v5wmy.

**Fig. 9.**
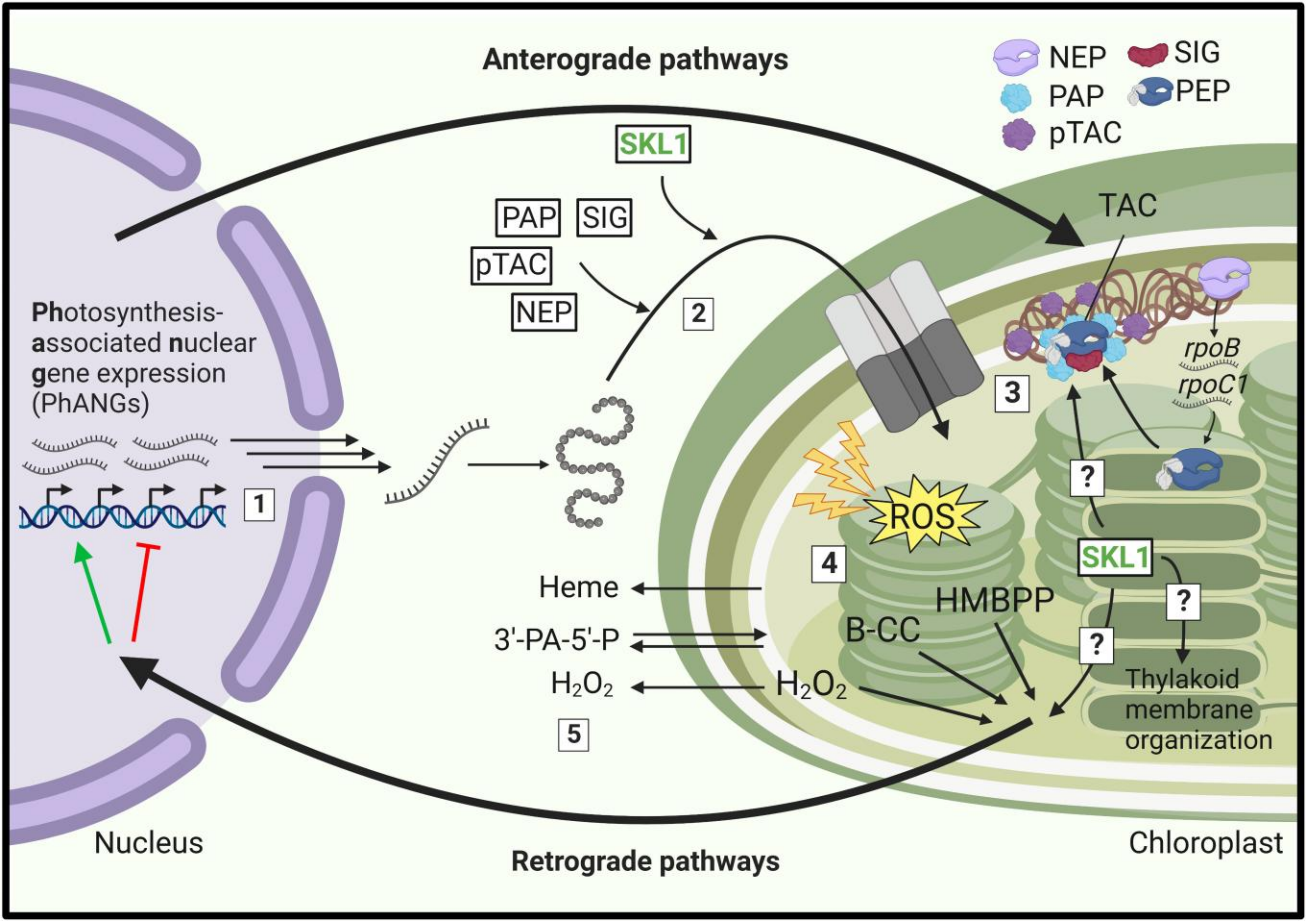
SKL1 is required for chloroplast biogenesis in plants. a) [1] The coordination of nuclear gene expression associated with chloroplast biogenesis and homeostasis involves a number of forward (anterograde) and reverse (retrograde) signaling pathways. [2, 3] Gene products destined for the chloroplast act within critical roles as early biogenesis factors, which include proteins necessary to establish and regulate plastid gene expression at the transcriptionally active chromosome (TAC). Appropriate plastid gene expression is regulated by nuclear-encoded gene products, whereas many plastid-encoded gene products are necessary to establish the photosynthetic apparatus and thylakoid membrane networks. [4, 5] Photosynthesis incurs reactive oxygen species that generate secondary metabolite and small molecule messengers and are relayed within retrograde pathways in the cell to modulate nuclear gene expression, and may also include secondary protein messengers (not depicted). SKL1 is a nuclear-encoded gene product that plays an essential role for chloroplast biogenesis in plants through an unknown mechanism. The role of SKL1 may involve previously established processes that are necessary for chloroplast biogenesis and whose genetic disruptions also result in pigment defects. NEP, nuclear-encoded plastid RNA polymerase; PEP, plastid-encoded plastid RNA polymerase; PAP, PEP-associated protein; SIG, sigma factor; pTAC, protein of TAC; B-CC, β-cyclocitral; HMBPP, hydroxymethylbutenyl diphosphate; 3′-PA-5′-P, adenosine-3′-phosphate-5′-phosphosulfate. Figure created with BioRender, https://www.BioRender.com/tq5ibei.

Early land plants faced a multitude of environmental pressures that were associated with their evolution, including a successful transition, colonization, and diversification in a terrestrial environment (Fig. 8; Donoghue et al. 2021; Schreiber et al. 2022). The acquisition of plastids during a primary symbiosis event leading to the evolution of photosynthetic algae was insufficient to fully support a lifestyle suited for land (Umen 2014; de Vries and Archibald 2018). However, the rapid transfer of genetic controls from the plastid to the nucleus was a key feature in the initial establishment of this endosymbiotic relationship (Martin et al. 1998; Keeling and Palmer 2008). The further development of genetic control mechanisms to regulate chloroplast biogenesis in ancestral land plants was critical in a terrestrial environment. We envision that SKL1 is one such example of this evolved mechanism of control in our proposed model. The acquisition of SKL1 as a nuclear-encoded, chloroplast-localized gene product and its retention in land plants indicates an ancient role in chloroplast biogenesis. As such, this provided additional controls for ancestral plant nuclear genomes to regulate chloroplast biogenesis in a manner that was desynchronized and independent from the endogenous plastid division machinery. This mechanism was likely integrated within networks of other anterograde and retrograde signaling pathways that have been extensively studied in relation to chloroplast biogenesis in plants (Fig. 9; Richter et al. 2023), some of which coincide with the transition to land (Raval et al. 2024). These events facilitated the adoption of plant cells housing many chloroplasts (de Vries and Gould 2018) and support for complex and amenable photosynthetic apparatuses (Ostermeier et al. 2024; Perez-Boerema et al. 2024), which are key features of plant chloroplasts. It is likely that SKL1 is not acting independently in this biological process and involves additional supporting components. It is therefore highly interesting and motivating to identify a chloroplast developmental pathway that is conserved within land plants.

## Materials and Methods

### Cloning of MpSK and MpSKL1 Protein Expression Constructs

MpSK (Mp3g21830) and MpSKL1 (Mp6g03600) sequences were identified using the *M. polymorpha* genome database MarPolBase (Bowman et al. 2017) and were acquired using the *Arabidopsis* SK gene homologs as pBLAST search queries. Putative cTP sequences were predicted using ChloroP 1.1 webserver (Emanuelsson et al. 1999) in addition to multiple sequence alignments with other plant SK and SKL1 sequences. Multiple constructs for MpSK (Δ106, Δ113, Δ115, Δ119) and MpSKL1 (Δ70, Δ74, Δ76, Δ80) were cloned from Tak-2 cDNA into the protein expression vector pET28a-MOD (C-terminal His_6x_) and transformed into BL21-CodonPlus. Each construct was sequence verified at the Centre for the Analysis of Genome Function and Evolution (CAGEF). All expressed proteins were purified by Ni-NTA chromatography, of which the His_6x_ tag was cleaved by TEV protease, and were then subjected to kinetic analyses. All generated constructs qualitatively showed similar levels of protein solubility properties, of which the Δ113MpSK and Δ80MpSKL1 constructs were chosen for downstream analyses. Primers for the constructs generated are indicated in supplementary table S1a, Supplementary Material online.

### Shikimate Kinase Assays

In vitro determinations of SK activity were carried out by use of a continuous pyruvate kinase–lactate dehydrogenase (PK-LDH) coupled assay as described previously (Fucile et al. 2008) with minor modifications. In brief, ∼4 to 8 μg of recombinant protein was added to a 1-mL kinetics buffer containing 50 mM Tris–HCl pH 7.5, 200 mM NaCl, 1 mM phosphoenolpyruvate, 0.15 mM NADH, 2.5 mM ATP, 1 mM MgCl_2_, and 2 μL (∼1.6 U/2.3 U) PK/LDH (Sigma-Aldrich). To establish Michaelis–Menten saturation kinetics, the concentration of shikimate substrate was varied as indicated (0 to 3 mM). Reactions were carried out at a constant 30 °C, where velocity measurements (μM NADH s^−1^) were determined based on the oxidation of NADH observed at 340 nm (*ε* = 6.22 × 10^3^ M^−1 ^cm^−1^). Kinetics were monitored using a Varian Cary 50 UV–visible spectrophotometer. Saturation curves were generated using GraphPad PRISM 9 for which determinations of kinetics constants were calculated.

### Confocal Microscopy Imaging and MpSKL1-RFP Localization Analysis

Maximum projection images were acquired on a Leica TCS SP5 confocal microscope at 10× or 20× objective magnification, with the use of an argon laser set to a power of 20%. The scanning mode was acquired using a bidirectional X at 200 Hz, 1,024 × 1,024 resolution. Excitation wavelengths (AOTF): mGFP5 (488 nm—30% intensity), tagRFP (543 nm—31% intensity). Detection windows: mGFP5 (500 to 550 nm), tagRFP (578 to 600 nm), and chlorophyll autofluorescence (650 to 700 nm). Detection of chlorophyll autofluorescence was carried out using the same excitation laser wavelength and intensity for the given reporter, where appropriate.

For localization analysis in *M. polymorpha*, MpSKL1 was C-terminally fused with tagRFP observed in a Tak-2 wild-type background. In brief, the coding sequence of *MpSKL1* was polymerase chain reaction (PCR) amplified using Gateway-compatible attB1 and attB2 sites, consisting of read-through nucleotides in the attB2 site. The resulting product was introduced into pDONR207 by BP Clonase II recombination. Entry clones were sequence verified at CAGEF before being subjected to recombination into the destination vector by LR Clonase II. The pMpGWB127 destination vector consisted of a C-terminally fused tagRFP reporter with a hygromycin resistance selectable marker (Ishizaki et al. 2015) driven by the *M. polymorpha* EF1α promoter (Althoff et al. 2014). The final expression clones were introduced into *A. tumefaciens* (GV2260) and then transformed into Tak-2 wild type, as described previously. Primers for the constructs generated are indicated in supplementary table S1b, Supplementary Material online.

### Liverwort and *Arabidopsis* Plant Growth Conditions

Col-0 ecotype and heterozygous *skl1*  *−*  *8 A. thaliana* plants were grown on standard half-strength Murashige and Skoog (MS) media (0.5×, 2.5 mM MES, pH 5.7) supplemented with 1.5% sucrose. Seeds were surface sterilized with ethanol and imbibed for a minimum of 2 d in water at 4 °C in the dark before plating on media. Seedlings were germinated under 24-h light conditions (80 μmol m^−2^ s^−1^, 22 °C) for the times designated. *Marchantia polymorpha* Tak-2 ecotype was provided courtesy of Dr. Kimitsune Ishizaki. Gemmalings were grown on standard half-strength Gamborg B5 media (0.5×, 2.5 mM MES, pH 5.7) supplemented with or without 1% sucrose in the same light conditions described previously for the durations designated in each experiment.

### cDNA Synthesis and MpSKL1 Semiquantitative Expression Analysis

Total RNA was extracted from *A. thaliana* Col-0 ecotype and *M. polymorpha* Tak-2 ecotype using TRI reagent (Sigma-Aldrich) according to manufacturer's instructions, utilizing ∼5 to 20 mg of dry weight tissue for extractions. cDNA synthesis was carried out using 1 μg of total RNA using SensiFast cDNA synthesis kit (Bioline) according to manufacturer's instructions. Subsequent PCR amplification was carried out using 2 μL of cDNA using Q5 polymerase (New England Biolabs). For semiquantitative RT-PCR analysis of SKL1 gene expression in *M. polymorpha*, gemmalings were grown for ∼1 month in the absence of sucrose before total RNA was isolated as previously indicated. Primers specific for the *MpSKL1* locus were utilized in addition to the house-keeping gene *MpEF1α* (Mp3g23400) and are viewed in supplementary table S1c, Supplementary Material online.

### Agrobacterium-Mediated Transformation of *M. polymorpha*

Transformations of *M. polymorpha* Tak-2 were carried out using AgarTrap agrobacterium-mediated procedures (Tsuboyama et al. 2018) with minor modifications. In brief, gemma was spread on 0.5× Gamborg plates supplemented with 1.0% sucrose and were grown for 4 d before being subjected to coculture with *Agrobacterium* (GV2260) containing Gateway expression plasmids with the appropriate antibiotics. Before coculture, *Agrobacterium* cells were resuspended in transformation medium (10 mM MgCl_2_, 10 mM MES pH 5.7, 0.01% Tween 20, and 150 μM acetosyringone) to an OD_600_ of 0.5, of which 1 mL of resuspended cells was added to the 4-d-old gemmalings. Excess transformation medium was removed from the plate, after which the cocultured plates were sealed with parafilm and grown in the dark for 2 d under normal growth conditions. The *Agrobacterium* cells were then rinsed with two rounds of 4 mL washes of sterile water, and then, 1 mL of selection medium (100 μg/mL hygromycin or 50 μg/mL G418, where relevant, with 1 mg/mL cefotaxime) was added to the plate. The final concentrations of antibiotics were diluted ∼10-fold in the plate, which contained 10 mL of solidified medium. The plates were sealed with parafilm for 2 to 3 weeks before T1 transformants were transferred to fresh 0.5× Gamborg plates supplemented with 1.0% sucrose, 10 μg/mL hygromycin or 5 μg/mL G418, and 100 μg/mL cefotaxime. Subsequent generations isolated from gemma cups were propagated without the use of antibiotics.

### Mutagenesis of MpSKL1 by CRISPR/Cas9 Mutagenesis

Mutants of the *MpSKL1* locus were generated based on procedures described by Sugano et al. (2018) using CRISPR/Cas9 mutagenesis. Cloning of the expression construct was carried out using a Gateway-compatible approach. First, the pMpGE_En03 entry clone harboring an expression cassette, driven by the MpU6-1 promoter, contained the gRNA backbone with an interchangeable sgRNA target sequence. To modify the sgRNA target sequence, two self-complementary oligonucleotides contained the 20-bp sgRNA sequence with a 4-bp vector-specific overhang on both the 5′ and 3′ ends to ligate into the BsaI-digested entry clone for a seamless fusion. Top scoring sgRNA sequences were determined using the CRISPR-P 2.0 webserver for the *MpSKL1* locus (Liu et al. 2017). The sgRNA2 guide was located in the 5′ UTR, whereas the sgRNA3 guide targeted a region lying shortly after the encoded predicted cTP. Entry clones were sequence verified at CAGEF before being subjected to recombination using LR Clonase II into the all-in-one pMpGE010 destination vector, containing an *Arabidopsis* codon-optimized Cas9 driven by the *MpEF1α* promoter. The final pMpGE010-sgRNA2 and pMpGE010-sgRNA3 expression vectors were transformed into Tak-2 ecotype using the previously described methods. Isolation of the *skl1*  *+*  *1* mutant was derived from the use of *Agrobacterium* coinoculation of both sgRNA2 + 3 expression vectors, whereas *skl1*  *+*  *9* was isolated from the use of the sgRNA3 expression vector only. The isolated mutants were sequence verified at their targeted sites, where appropriate, of which two independent sequencing primers were used to verify the indels. Oligomers utilized for the constructs generated are indicated in supplementary table S1d, Supplementary Material online.

### Cloning of AtcTP-Δ80MpSKL1-GFP and AtcTP-Δ113MpSK-GFP Fusion Constructs

To generate the two-fragment AtcTP-Δ80MpSKL1 and AtcTP-Δ113MpSK fusion construct, an overlap extension PCR method (Bryksin and Matsumura 2010) was used to create an in-frame fusion of the first DNA fragment consisting of *A. thaliana* SKL1 cTP (AtcTP) to the second DNA fragment consisting of *M. polymorpha* SKL1 or SK with its endogenous cTP removed (Δ80MpSKL1 or Δ113MpSK, respectively). Approximate cTP lengths for the included proteins were predicted from ChloroP 1.1 server (Emanuelsson et al. 1999) and by use of sequence alignments of multiple plant SK and SKL1 sequences. On the 5′ end of the second DNA fragment, a 20-bp sequence overlap with the 3′ end of the AtcTP fragment was added to facilitate overlap extension by Q5 polymerase. Individual fragments were first amplified from previously verified Δ80MpSKL1 or Δ113MpSK constructs, or *A. thaliana* cDNA for the AtcTP fragment (56 amino acids in length). To carry out the overlap extension, a PCR reaction was conducted using ∼10 to 20 ng of both DNA fragments with the forward primer of AtcTP and the reverse primer of MpSKL1 or MpSK. The two-fragment fusion product was gel excised before cloning into a shuttle vector using XbaI and BamHI. Isolated clones were sequence verified for the correct in-frame fusion before undergoing amplification to add on attB1 and attB2 Gateway-compatible sites. The two-fragment fusion was subjected to recombination into pDONR207 by BP Clonase II and was sequence verified before undergoing recombination into the destination vector pEarleygate103 (Earley et al. 2006) by LR Clonase II containing a C-terminally fused mGFP5 tag driven by an enhanced 35S promoter. The final expression clones were introduced into A. *tumefaciens* (GV3101) before being transformed into *A. thaliana* by floral dip. Primers for the constructs generated are indicated in supplementary table S1e, Supplementary Material online.

### Genotyping of *skl1−8* T-DNA and Ectopic Transgenes

Transgenic seedlings isolated from AtcTP-Δ80MpSKL1-GFP and AtcTP-Δ113MpSK-GFP transformed plants within the T2 generation were individually isolated and subjected to genotyping analysis. Genomic DNA was isolated for whole seedlings ∼1.5 to 2.0 weeks old using cetyltrimethyl ammonium bromide (CTAB) DNA extraction buffer (2.0% CTAB, 1.2 M NaCl, 20 mM EDTA, 100 mM Tris–HCl pH 8.0) followed by a chloroform extraction (24:1 chloroform/isoamyl alcohol). The DNA-containing aqueous layer was precipitated with 1:1 addition of 100% isopropanol, followed by washing the DNA pellet with 70% ethanol, and was dissolved in DNase-free water. Subsequent PCR (∼75 to 100 ng gDNA) was conducted for specificity of the T-DNA insertions of the *AtSKL1* locus (*skl1*  *−*  *8*) and the presence of the ectopic expression construct. To genotype for *skl1*  *−*  *8* insertions, two PCR reactions were conducted: (i) T-DNA-specific forward primer Lba1- and SKL1-specific reverse primer (SKL1-R) and (ii) SKL1-specific primer pair (AtSKL1-F and SKL1-R), with the forward primer containing flanking the T-DNA insertion. Homozygous *skl1*  *−*  *8* mutants contain only an amplified band in the T-DNA PCR, whereas heterozygous *skl1*  *−*  *8* mutants contain the T-DNA and the SKL1-specific band. To genotype the transgenic ectopic expression constructs, primers previously designed for the protein expression constructs were utilized. Primers used for genotyping are listed in supplementary table S1f, Supplementary Material online.

### Chlorophyll and Total Carotenoids Quantifications

Quantification of chlorophyll and total carotenoids content from both *M. polymorpha* and *A. thaliana* tissue was derived from Lichtenthaler and Wellburn (1983). In brief, 10 to 50 mg of tissue from seedlings at the ages indicated was harvested from media-containing agar plates and was suspended in 1 mL of 96% ethanol. The tissue was gently rocked for 3 h in the dark to prevent any light-induced pigment degradation. Subsequently, the samples were centrifuged at 14,000 × *g* for ∼5 min to pellet the tissue where the pigment-containing supernatant was isolated. The absorbance spectra (300 to 800 nm) of the extracted pigments were monitored in a Varian Cary 50 UV–Vis Spectrophotometer (Agilent Technologies). Samples were analyzed in three biological replicates, of which the SDs are indicated.

### Transmission Electron Microscopy

Gemmae from Tak-2 and *skl1*  *+*  *1* were grown on standard Gamborg media plates for 13 d before fixation in 3% glutaraldehyde in 0.1 M Sorenson's phosphate buffer (pH 7.4) with a subsequent brief vacuum infiltration. Tissue was excised near the pale green elongation zones of phenotypic tissue from *skl1*  *+*  *1* with comparison to similar regions in Tak-2 preparation. The samples were rinsed and postfixed in 1% OsO_4_ (Electron Microscopy Sciences) for 1 h. After that, the tissue was rinsed again with 0.1 M Sorenson's phosphate buffer, dehydrated through an ascending ethanol series, and then infiltrated with and embedded in modified Spurr's resin. From the area of interest, identified by thick sectioning, ultrathin sections (90 to 100 nm) were cut with a Leica UC6 ultramicrotome. Thin sections were stained with uranyl-less and lead citrate then examined with the Hitachi HT7700 transmission electron microscope. For *Arabidopsis* preparations, both Col-0 and homozygous *skl1*  *−*  *8* seedlings were grown on standard MS media for 10 d before glutaraldehyde fixation, while the AtcTP-Δ80MpSKL1-GFP seedlings were fixed at 12 d. Excised tissue was isolated near the central vein from both the cotyledons and true leaves and was fixed and prepared as described previously.

### Structural Modeling and Ligand Docking

The 3D structures of MpSK and MpSKL1 were modeled using the Robetta RoseTTAFold deep learning algorithm prediction service (Baek et al. 2021). The sequences were submitted with the N-terminal transit peptides removed (Δ113MpSK, Δ80MpSKL1). Additionally, MpSK was modeled with its LID domain removed for clarity of the nucleotide and shikimate binding pockets. The models were compared with the *A. thaliana* crystal structure (PDB ID: 3nwj) in addition to the various liganded *M. tuberculosis* crystal structures (Hartmann et al. 2006). Residue placements within the shikimate binding pocket were validated for consistency between AtSK2 and MtSK with bound shikimate (PDB ID: 2iyx) before visualization within the MpSK model. For the nucleotide binding domain, the dissimilarity of the AB-loop between MtSK and AtSK2 led us to visualize the nucleotide pocket by docking analysis using AutoDock Vina (Morris et al. 2009; Trott and Olson 2010). High-confidence positioning of ATP within AtSK2 and MpSK was determined based on the similarity of the participating hydrogen bonds within the P-loop of MtSK (PDB ID: 2iyw). Calculation of the electrostatic surface charges for the crystal structures and models was determined using the Adaptive Poisson-Boltzmann Solver (APBS) extension in PyMOL 2.5 (Jurrus et al. 2018).

### Phylogenetic Reconstruction of SKL1 and SK Orthologs

Acquisition of putative SKL1 and SK amino acid sequences within the land plant kingdom was gathered from the One Thousand Plants Project database (v5) (Matasci et al. 2014) derived from the One Thousand Plant Transcriptomes Initiative (Leebens-Mack et al. 2019). pBLAST query searches were carried out using MpSKL1 and MpSK. Initial species list parsing of the sequences was carried out using the four-letter identifiers provided by the OneKP project. Sequences containing the same four-letter identifier with the same amino acid sequence were treated as duplicates and were removed. Alternatively, sequences containing different four-letter identifiers with the same amino acid sequence were treated as unique sequences and were retained. The remaining sequences were subjected to multiple sequence alignment by ClustalOmega (Sievers et al. 2011) and were analyzed for incomplete, highly truncated, or contaminating SKL2 sequences, which were removed. Phylogenetic analysis of the remaining sequences was conducted using MEGA X (Kumar et al. 2018) by the neighbor-joining method using a Poisson model of amino acid substitution with 1,000 bootstrap replicates. The Interactive Tree of Life (iTOL) editor (v5) (Letunic and Bork 2021) was utilized to visualize the tree and annotated according to the major plant clades, as designated by the four-letter identifiers, and were further annotated using Inkscape. Clade groupings were generated for eudicots (core eudicots, rosids, asterids), monocots (monocots, commelinids), and basal angiosperms (basalmost angiosperms, magnoliids, and basal eudicots). Sequences that showed deviation from the conserved RPLL consensus motif for SK enzymes were annotated as putative SKL1 sequences. Consensus sequences were visualized using WebLogo (Crooks et al. 2004), and multiple sequence alignments were annotated using JalView2 (Waterhouse et al. 2009). The full list of amino acid sequences and sorting of the plant lineages from the OneKP project is viewed in supplementary table S2, Supplementary Material online.

### Analysis of Positive Selection by Codon Substitution Models

Analysis of positive selection for SK and SKL1 was conducted using the PAML4.9 package with codon-based measures of positive selection (codeml) (Yang 2007). Nucleotide sequences corresponding to the coding sequences for SK and SKL1 were gathered from the OneKP database, with a focus on collecting the identified sequences from the phylogenetic reconstruction analysis, where high-quality nucleotide sequences were available. Nucleotides encoding the N-terminal chloroplast peptide were removed from each of the sequences. The sequences were aligned using codon-based alignments in MEGA X by MUSCLE, and maximum likelihood (ML) trees were generated with 250 bootstrap replicates (TN93 + G). Various evolutionary models were estimated, including random-site model (model = 0, NSsites = 0, 1, 2, 3, 7, 8, 22), branch-site model (model = 2, NSsites = 2), and clade model C (model = 3, NSsites = 2). For the calculation of each respective nested null model, ω was fixed to 1.0, and the likelihood ratio test was determined for each estimation. Due to the large number of sequences, estimations of the synonymous (*d*_S_) and nonsynonymous (*d*_N_) rates, and other parameters and statistics were made using subgroupings of the plant lineages, as it was not feasible for the program to process all sequences within a single run due to large computation times. Therefore, evolutionary model estimates were applied to each plant lineage separately (liverworts, mosses, ferns, gymnosperms, angiosperms) or by overall representation (10 sequences from each lineage). The final lists of the nucleotide coding sequences are provided in supplementary table S3, Supplementary Material online. For branch-site and clade model C, SKL1 branches on the ML trees were specified as the foreground to allow *d*_N_/*d*_S_ (ω) to vary, with the SK sequences left as background branches. Full descriptions of the included calculations, parameters, and estimations for the evolutionary models are provided in supplementary tables S4 to S6, Supplementary Material online.

## Supplementary Material

msaf129_Supplementary_Data

## Data Availability

The data underlying this article are available in the article and in its online Supplementary material.
