# Supplementary material for: Shikimate Kinase-Like 1 Participates in an Ancient and Conserved Role Contributing to Chloroplast Biogenesis in Land Plants
Source: Mol Biol Evol. 2025 Jun 2;42(6):msaf129. doi: 10.1093/molbev/msaf129 (PMC12203367; doi:10.1093/molbev/msaf129)
Supplement: msaf129_Supplementary_Data [file msaf129_supplementary_data.zip › Supplemental Table 1.pdf]

Supplementary Table S1. List of primers used within this article.

A - List of primers used for cloning *Marchantia polymorpha* shikimate kinase (SK) and shikimate kinase-like 1 (SKL1) expression constructs for protein purification in pET28 vector series.

Primer Name	Primer Sequence (5' → 3')
Δ70 MpSKL1-NdeI-F	CAGCATATG TTTGCAGGGGCGGGAGG
Δ73 MpSKL1-NdeI-F	CAGCATATG GCGGGTGGCACTCCGGC
Δ76 MpSKL1-NdeI-F	CAGCATATG ACTCCGGCGAGGGACGAG
Δ80 MpSKL1-NdeI-F	CAGCATATG GACGAGGAGACATTGCTGAAGAAG
WT MpSKL1-Stop-BamHI-R	GCTGGATCC CTACGCTTTCTTTAGAACTCTATCG
WT MpSKL1-NoStop-BamHI-R	GCTGGATCC CGCTTTCTTTAGAACTCTATCG
Δ106 MpSK-NdeI-F	CAGCATATG GGAACATCTACAGACGAAAAGCAG
Δ113 MpSK-NdeI-F	CAGCATATG CAGTACTCTTTAGAGTTGCAGGAAGACG
Δ115 MpSK-NdeI-F	CAGCATATG TCTTTAGAGTTGCAGGAAGACGAGG
Δ119 MpSK-NdeI-F	CAGCATATG CAGGAAGACGAGGAGTTGCTC
WT MpSK-Stop-BamHI-R	GCTGGATCC CTAGTATTGTAGGCTGGGGTCCC
WT MpSK-NoStop-BamHI-R	GCTGGATCC GTATTGTAGGCTGGGGTCCC

Underlined portions of sequences indicate restriction sites. Green nucleotides indicate synonymous mismatches to alleviate strong hairpins during PCR amplification. Red nucleotides indicate the inclusion or removal of stop codons in the reverse primer, where appropriate.

B - List of primers used for generating Gateway-compatible PCR amplification products for cloning MpSKL1 into pDONR207.

Primer Name	Primer Sequence (5' → 3')
WT attB1 MpSKL1-F	GGGGACAAGTTTGTACAAAAAAGCAGGCTCC ATGGCAGTAATGGCTGGTC
WT attB2 MpSKL1-NoStop-R	GGGGACCACTTTGTACAAGAAAGCTGGGTA CGCTTTCTTTAGAACTCTATCGATC

Underlined portions indicate *attB1* or *attB2* sites in the forward and reverse primers, where appropriate. The green nucleotide indicates a synonymous mismatch to alleviate strong hairpins during PCR amplification. The *attB2* cyan nucleotide indicates a readthrough into the C-terminal fusion tag.

C - List of primers used for semi-quantitative expression analysis of Tak-2 wild type and *skl1+1* mutant gemmalings.

Primer Name	Primer Sequence (5' → 3')
MpEF1α-F	GCCTCGAGTAAAGCTTCGTG
MpEF1α-R	TCACTCTGGGTGTGAAGCAG
*Δ80 MpSKL1-NdeI-F	CAGCATATG GACGAGGAGACATTGCTGAAGAAG

*WT MpSKL1-Stop-BamHI-R	GCTGGATCC CTACGCTTTCTTTAGAACTCTATCG

*Primers previously indicated in Table A

D - List of oligomers used for assembly of the 20 bp sgRNA target sequences into the pMpGE_En03 donor vector.

**Oligomer Name**	**Oligomer Sequence (5' → 3')**
WT MpSKL1 Guide-2-F	**CTCG** ATATCTTTCGCCGTCCGCAG
WT MpSKL1 Guide-2-R	AAAC CTGCGGACGGCGAAAGATAT
WT MpSKL1 Guide-3-F	**CTCG** CCGTTCTCGACATACCCACG
WT MpSKL1 Guide-3-R	AAAC CGTGGGTATGTCGAGAACGG

Bolded sequences indicate the 5' overlap with the *MpU6-1* promoter, and underlined sequences indicate the 3' overlap with the gRNA backbone.

E - List of primers used to generate the AtcTP:Δ80MpSKL1 and AtcTP-Δ113MpSK-GFP fusion and subsequent cloning into pDONR207.

**Primer Name**	**Primer Sequence (5' → 3')**
SoeA-AtSKL1cTP-XbaI-F	GACTCTAGA ATGGAGATCTTCTCTGCGTCTG
SoeB-AtSKL1cTP-R	GAGAGACCGAGACGGA
SoeC-Δ80MpSKL1-F	**TAGCTCCGTCTCGGTCTCTC** GACGAGGAGACATTGCTGAAGAAG
SoeD-MpSKL1-NoStop-BamHI-R	GTCGGATCC CGCTTTCTTTAGAACTCTATC
SoeC-Δ113MpSK-F	**TAGCTCCGTCTCGGTCTCTC** CAGTACTCTTTAGAGTTGCAGGAAGAC
SoeD-MpSK-NoStop-BamHI-R	GTCGGATCC GTATTGTAGGCTGGGGTCCC
AtcTP-attB1-F	GGGGACAAGTTTGTACAAAAAAGCAGGCT ATGGAGATCTTCTCTGCGTCTG
MpSKL1-NoStop-attB2-R	GGGGACCACTTTGTACAAGAAAGCTGGGTC CGCTTTCTTTAGAACTCTATCGAT
MpSK-NoStop-attB2-R	GGGGACCACTTTGTACAAGAAAGCTGGGTC GTATTGTAGGCTGGGGTCCC

Bolded portions indicate the 5' 20 bp sequence overlap with the *AtSKL1* cTP contained within fragment two of the two-fragment fusion. Underlined portions indicate *attB1* or *attB2* sites or restriction enzyme cut sites, where appropriate. The *attB2* cyan nucleotide indicates a readthrough into the C-terminal fusion tag.

F - List of primers used for genotyping analysis of *skl1-8* T-DNA and transgenic overexpression constructs in *Arabidopsis thaliana*.

**Primer Name**	**Primer Sequence (5' → 3')**
1. *Δ80 MpSKL1-NdeI-F	CAGCATATG GACGAGGAGACATTGCTGAAGAAG
2. *WT MpSKL1-NoStop-BamHI-R	GCTGGATCC CGCTTTCTTTAGAACTCTATCG

3. *Δ113 MpSK-NdeI-F	CAGCATATG CAGTACTCTTTAGAGTTGCAGGAAGACG
4. *WT MpSK-NoStop-BamHI-R	GCTGGATCC GTATTGTAGGCTGGGGTCCC
5. LBa1-F	TGGTTCACGTAGTGGGCCATCG
6. AtSKL1-F	GGAAGCTTTTGGCTGAGG
7. SKL1-R	CTAGAAAGGTCGAGAAGCTTCTTCC

Primer pairs for genotyping seedlings: *MpSK* (1+2 – 676 bp) and *MpSKL1* (3+4 – 576 bp) transgenes, *skl1-8* T-DNA (5+7 - ~527 bp), and endogenous *AtSKL1* (6+7 – 1360 bp gDNA, 527 bp cDNA). *Primers previously indicated in Table A.
