# Supplementary figures and images for: Shikimate Kinase-Like 1 Participates in an Ancient and Conserved Role Contributing to Chloroplast Biogenesis in Land Plants
Source: Mol Biol Evol. 2025 Jun 2;42(6):msaf129. doi: 10.1093/molbev/msaf129 (PMC12203367; doi:10.1093/molbev/msaf129)

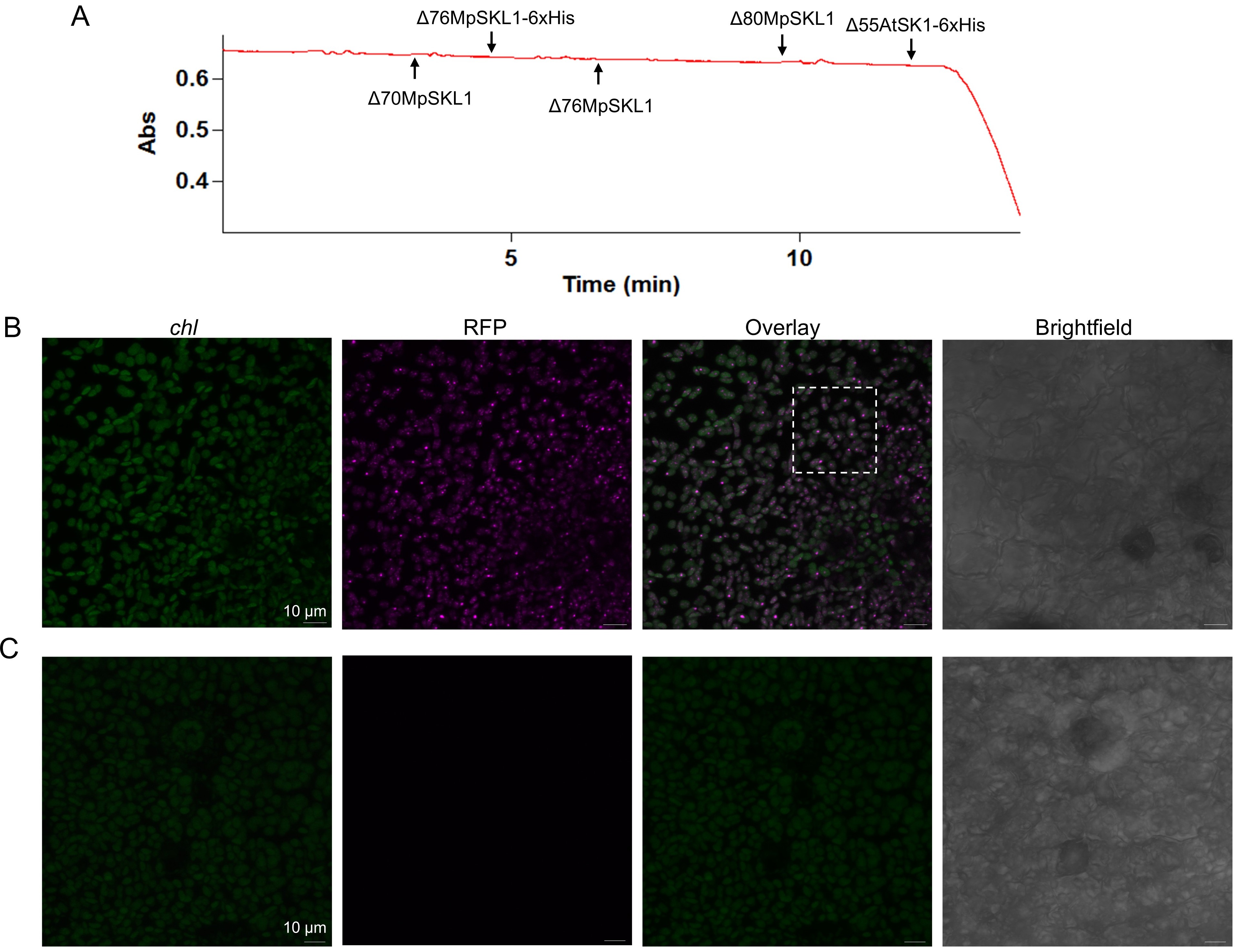

Supplement: msaf129_Supplementary_Data [file msaf129_supplementary_data.zip › Figure S1.jpg]

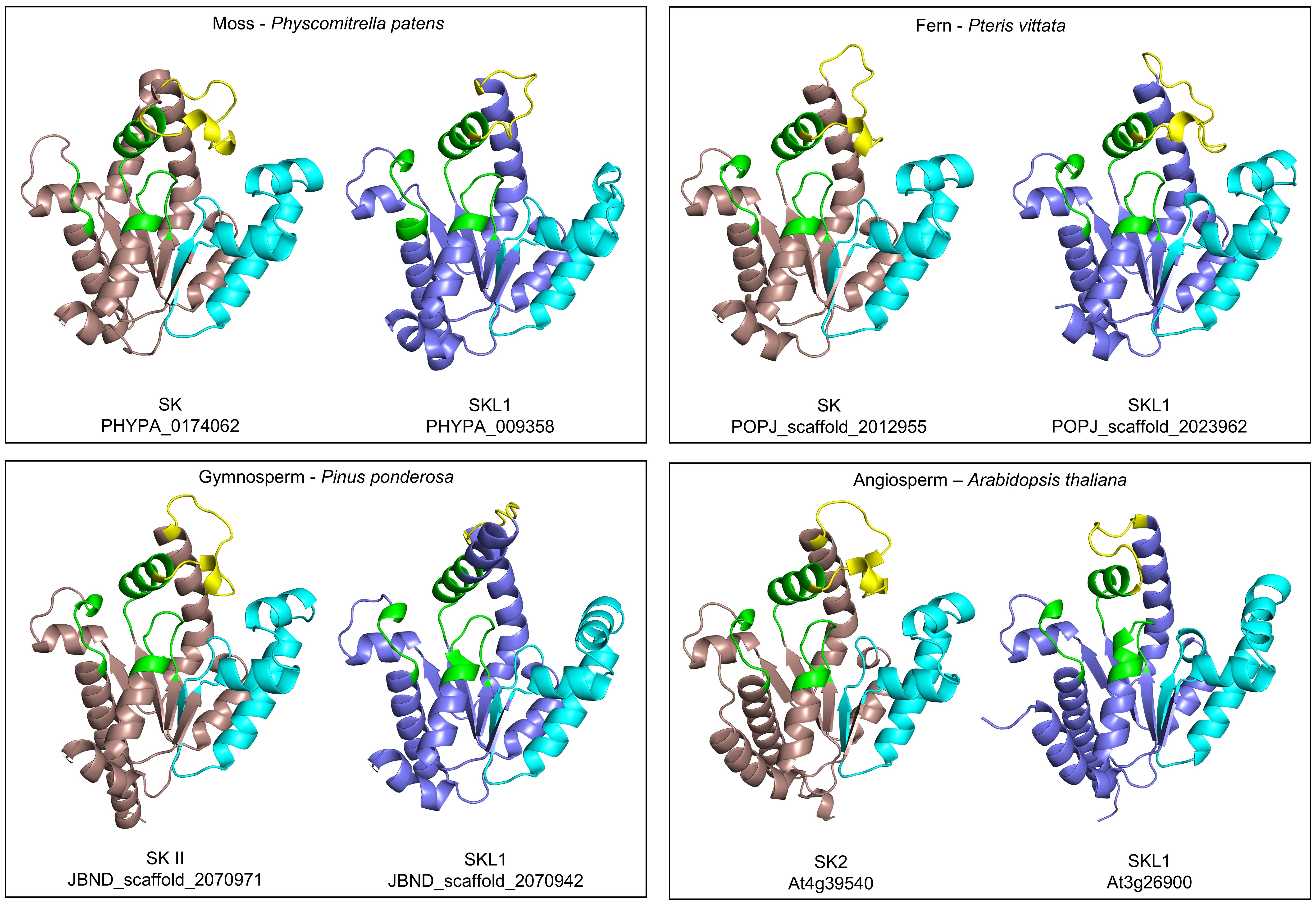

Supplement: msaf129_Supplementary_Data [file msaf129_supplementary_data.zip › Figure S10.JPG]

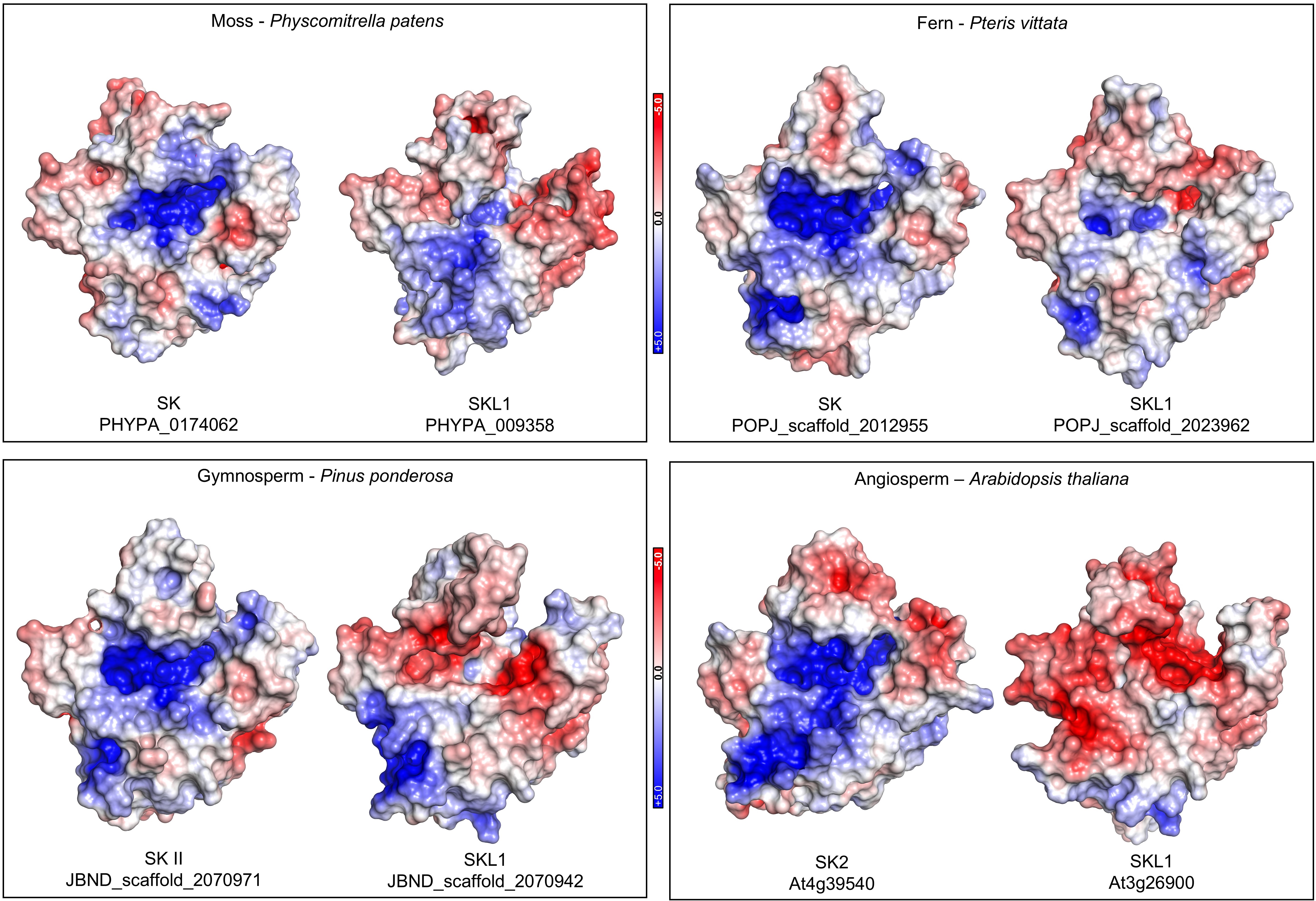

Supplement: msaf129_Supplementary_Data [file msaf129_supplementary_data.zip › Figure S11.JPG]

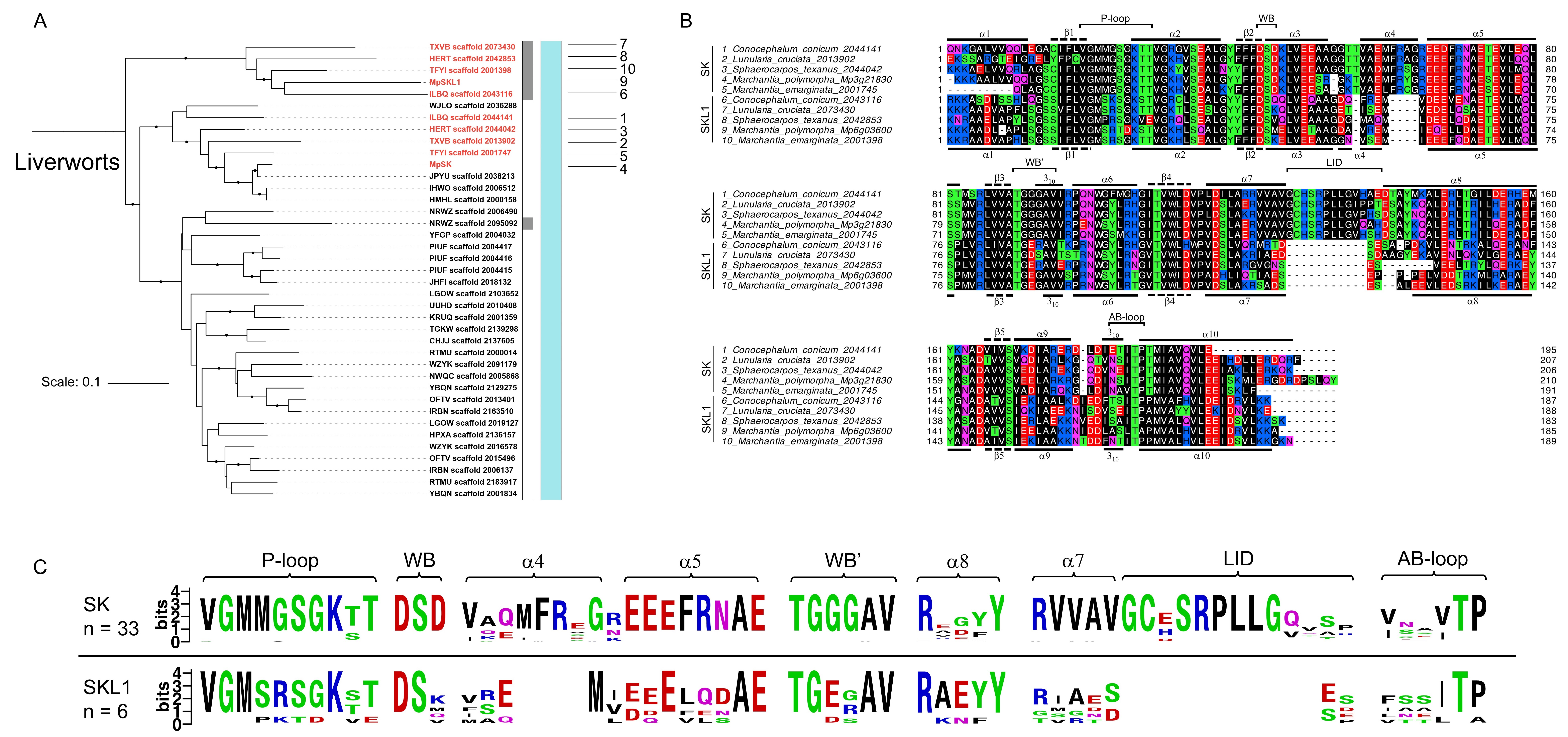

Supplement: msaf129_Supplementary_Data [file msaf129_supplementary_data.zip › Figure S12.JPG]

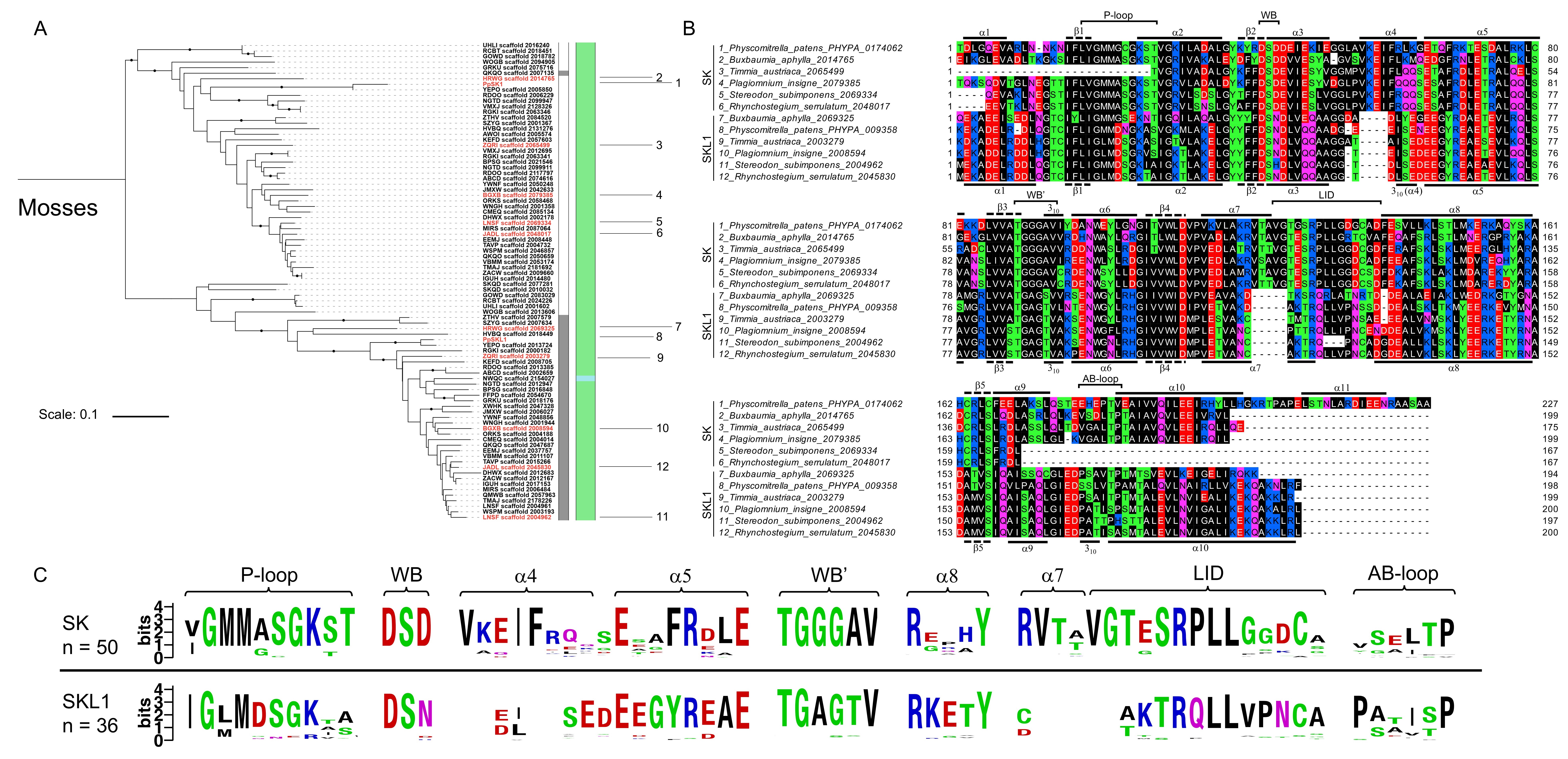

Supplement: msaf129_Supplementary_Data [file msaf129_supplementary_data.zip › Figure S13.JPG]

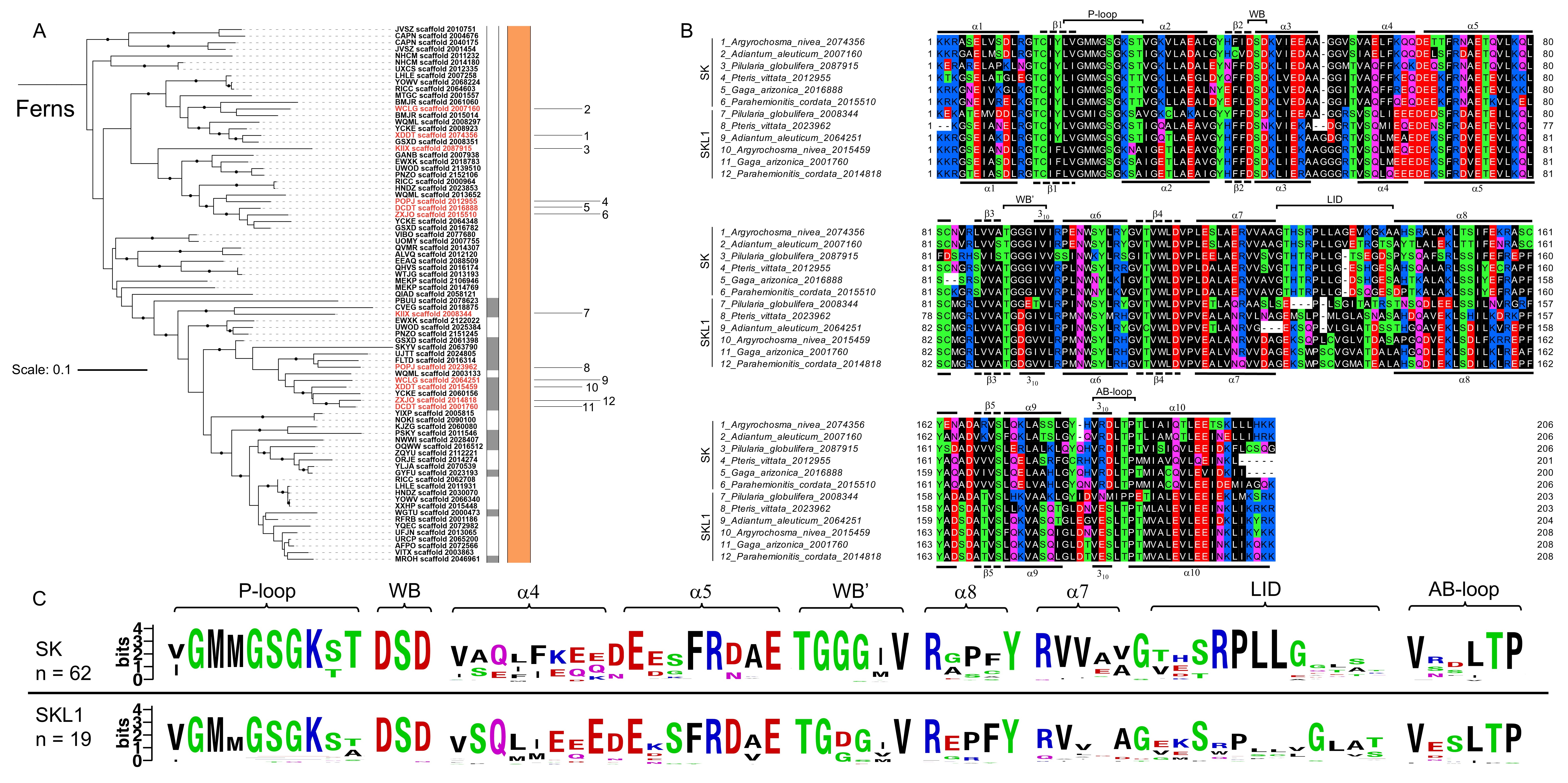

Supplement: msaf129_Supplementary_Data [file msaf129_supplementary_data.zip › Figure S14.JPG]

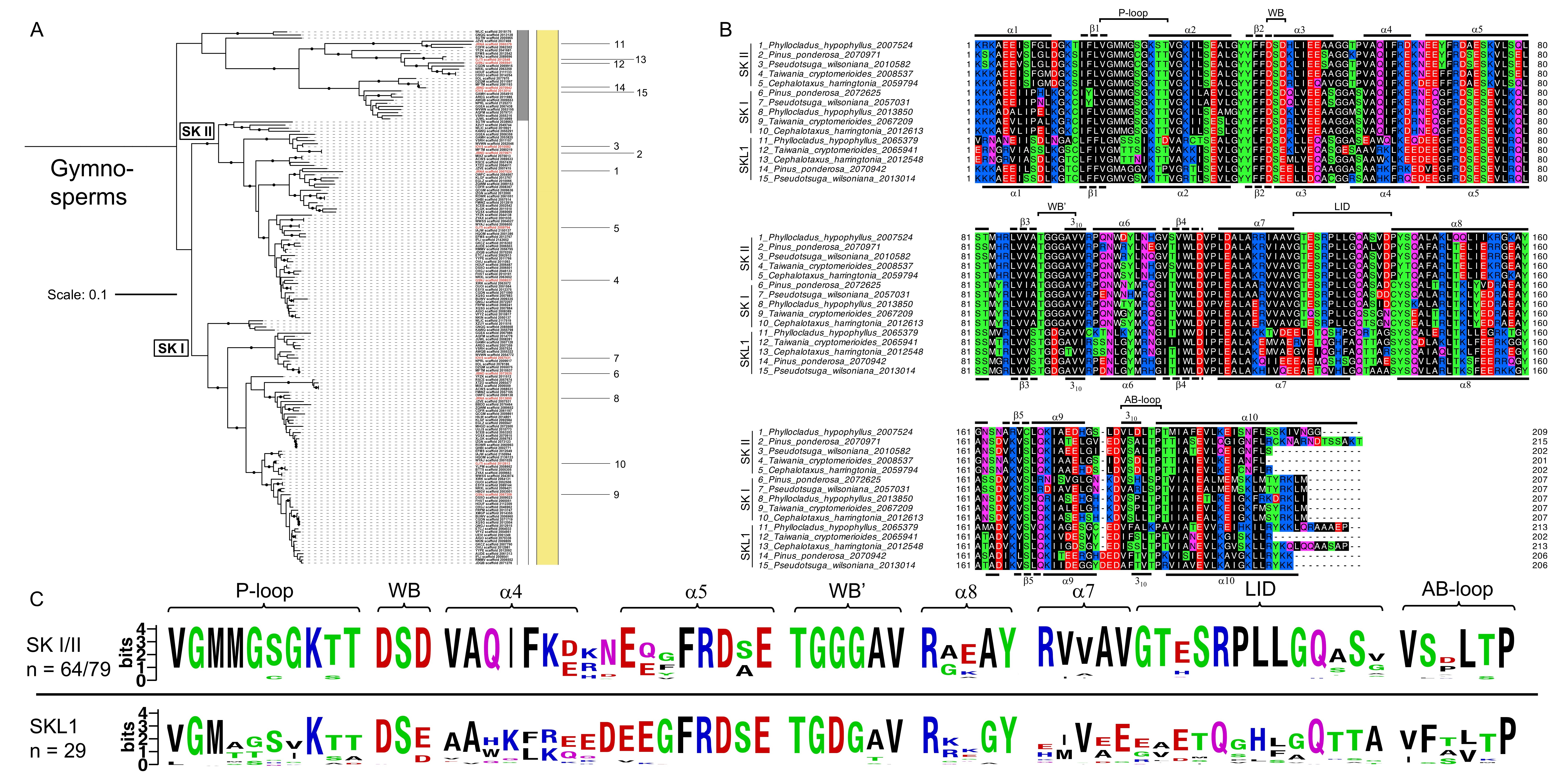

Supplement: msaf129_Supplementary_Data [file msaf129_supplementary_data.zip › Figure S15.JPG]

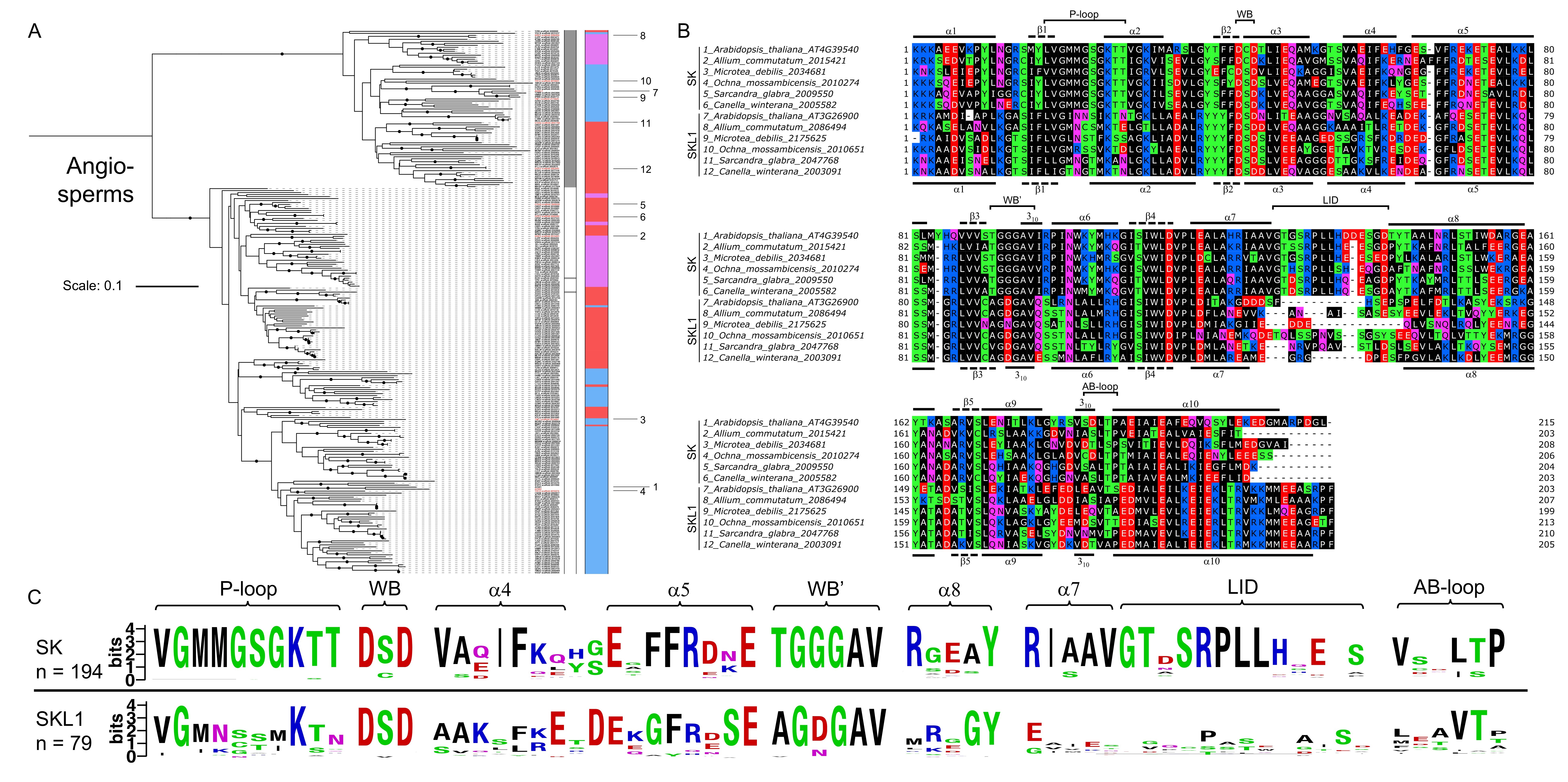

Supplement: msaf129_Supplementary_Data [file msaf129_supplementary_data.zip › Figure S16.JPG]

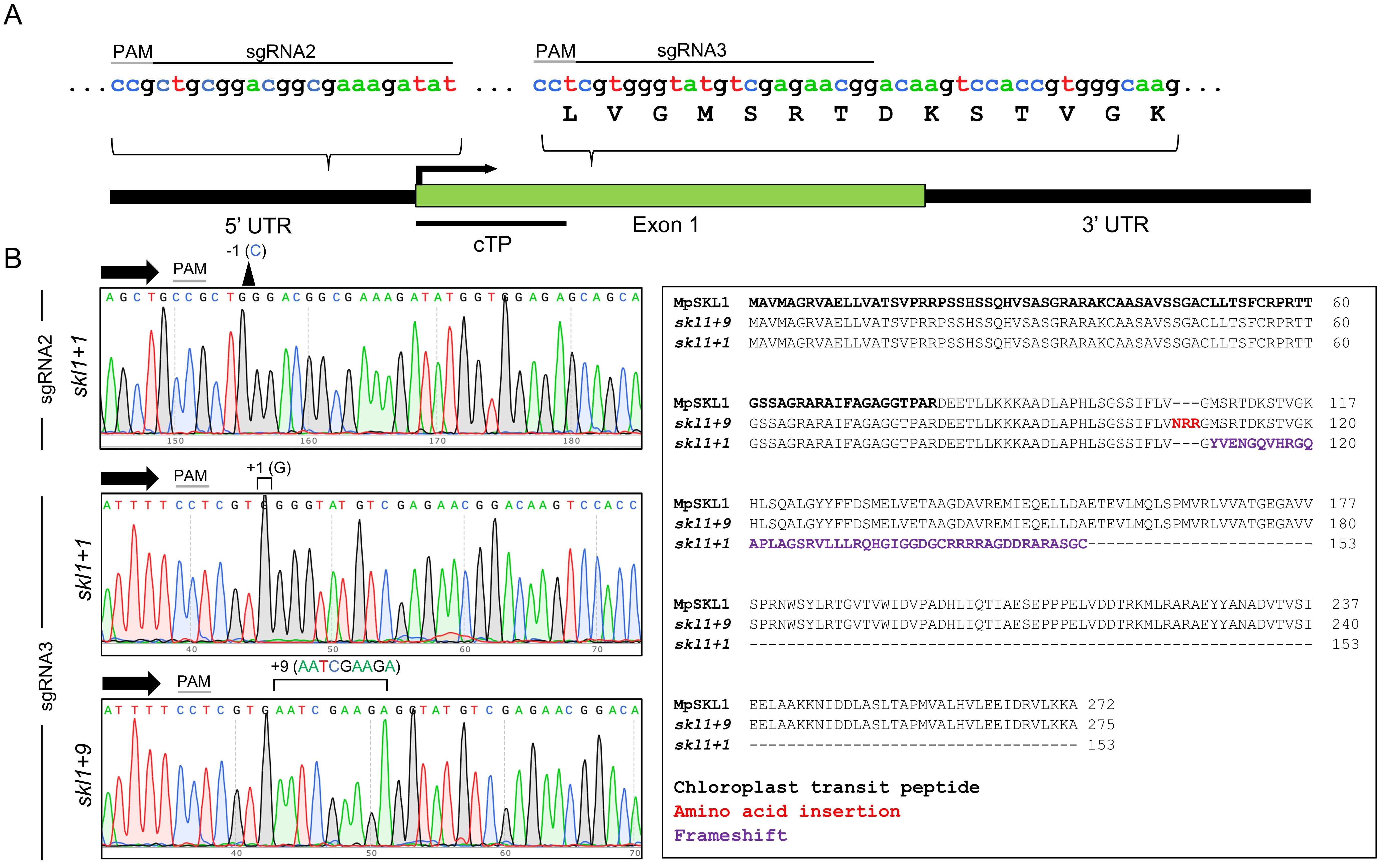

Supplement: msaf129_Supplementary_Data [file msaf129_supplementary_data.zip › Figure S2.JPG]

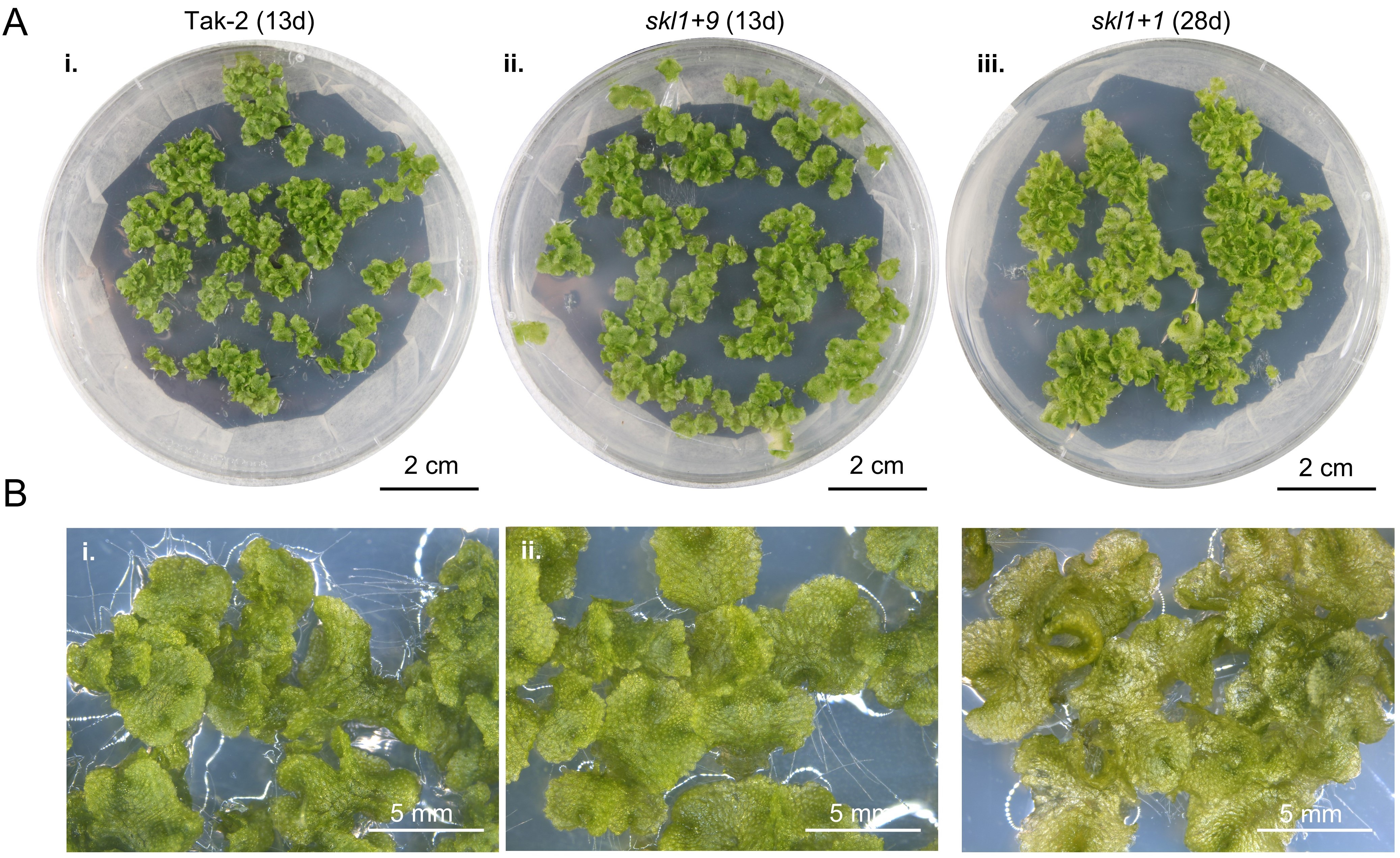

Supplement: msaf129_Supplementary_Data [file msaf129_supplementary_data.zip › Figure S3.JPG]

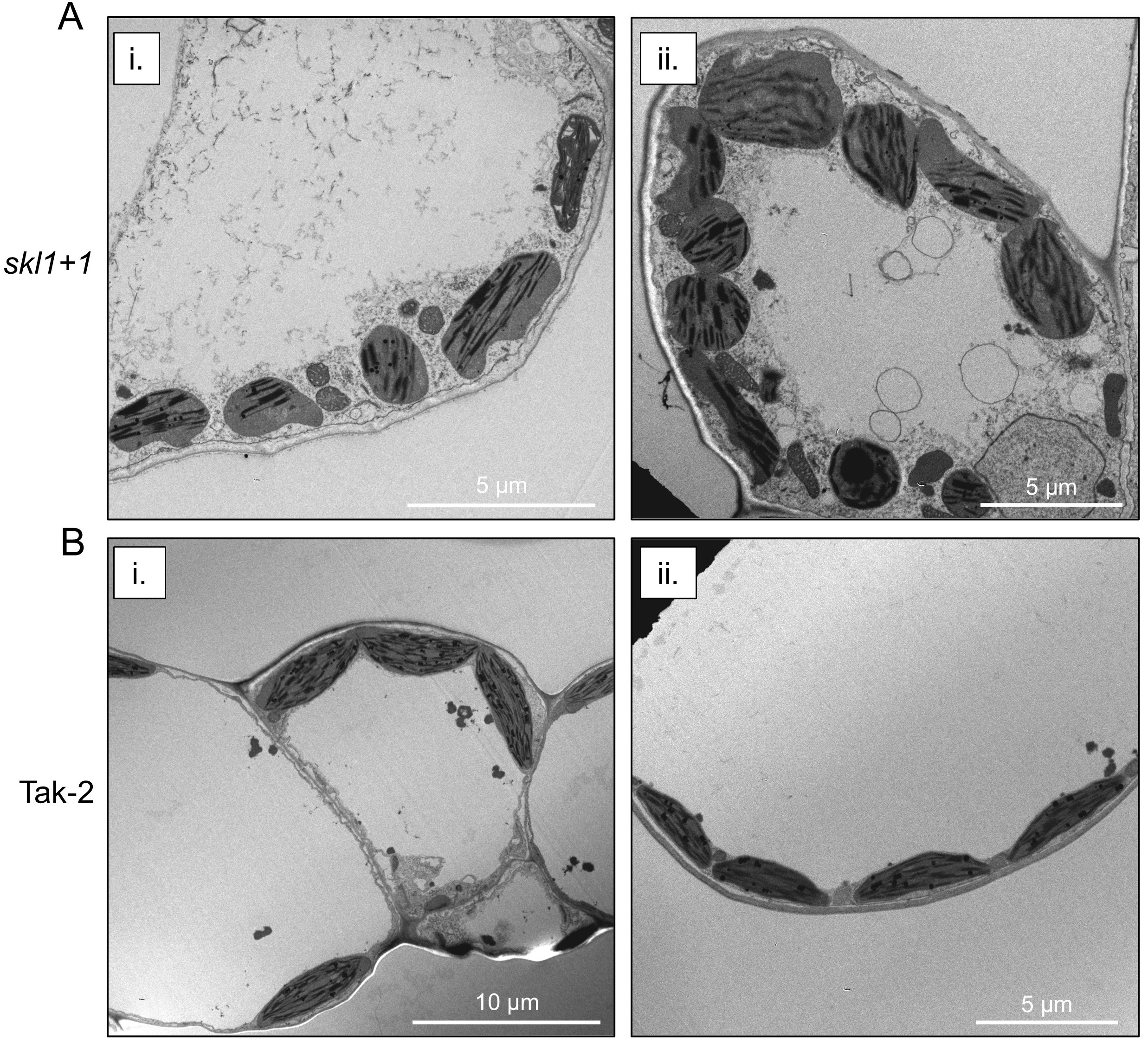

Supplement: msaf129_Supplementary_Data [file msaf129_supplementary_data.zip › Figure S4.JPG]

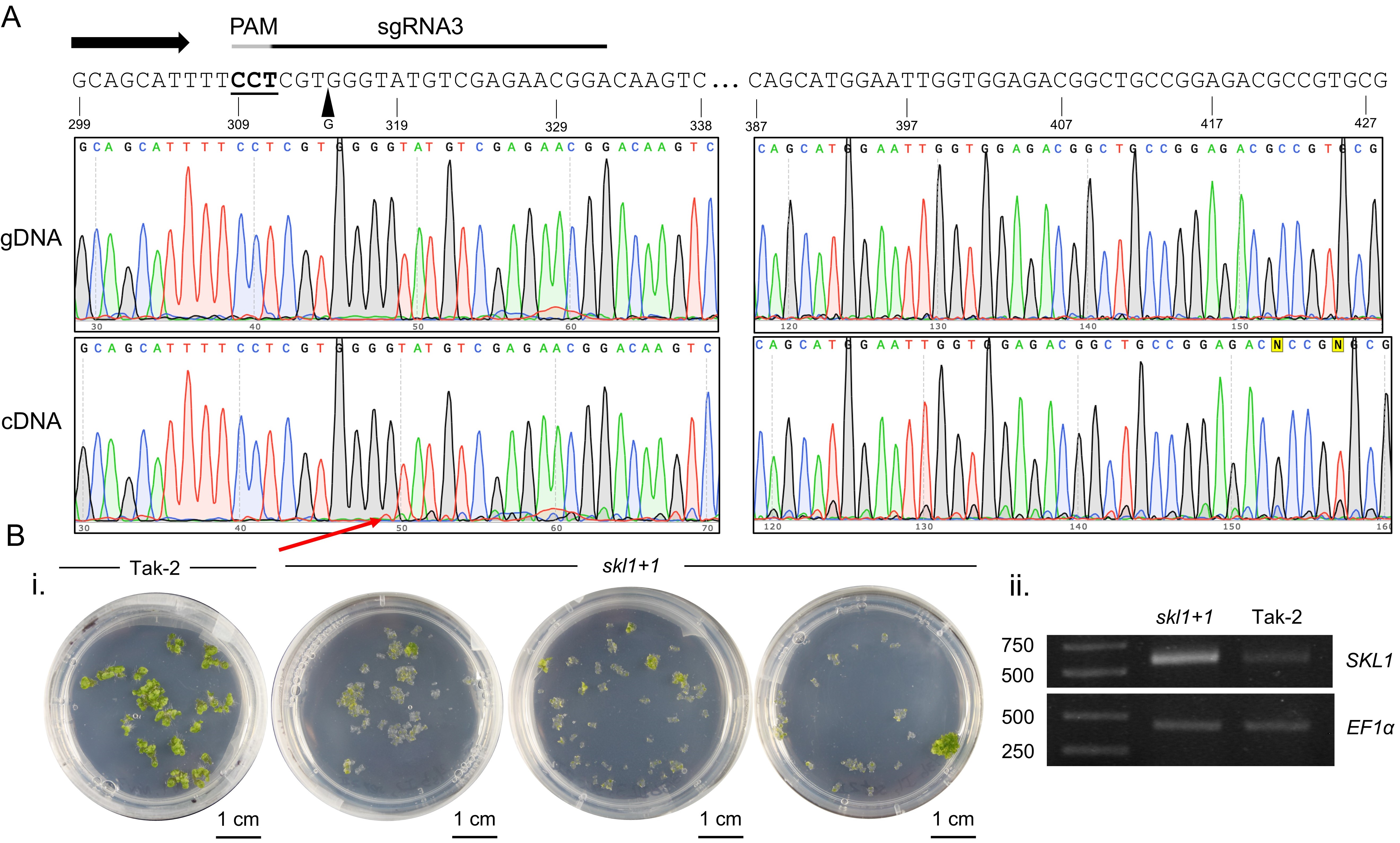

Supplement: msaf129_Supplementary_Data [file msaf129_supplementary_data.zip › Figure S5.JPG]

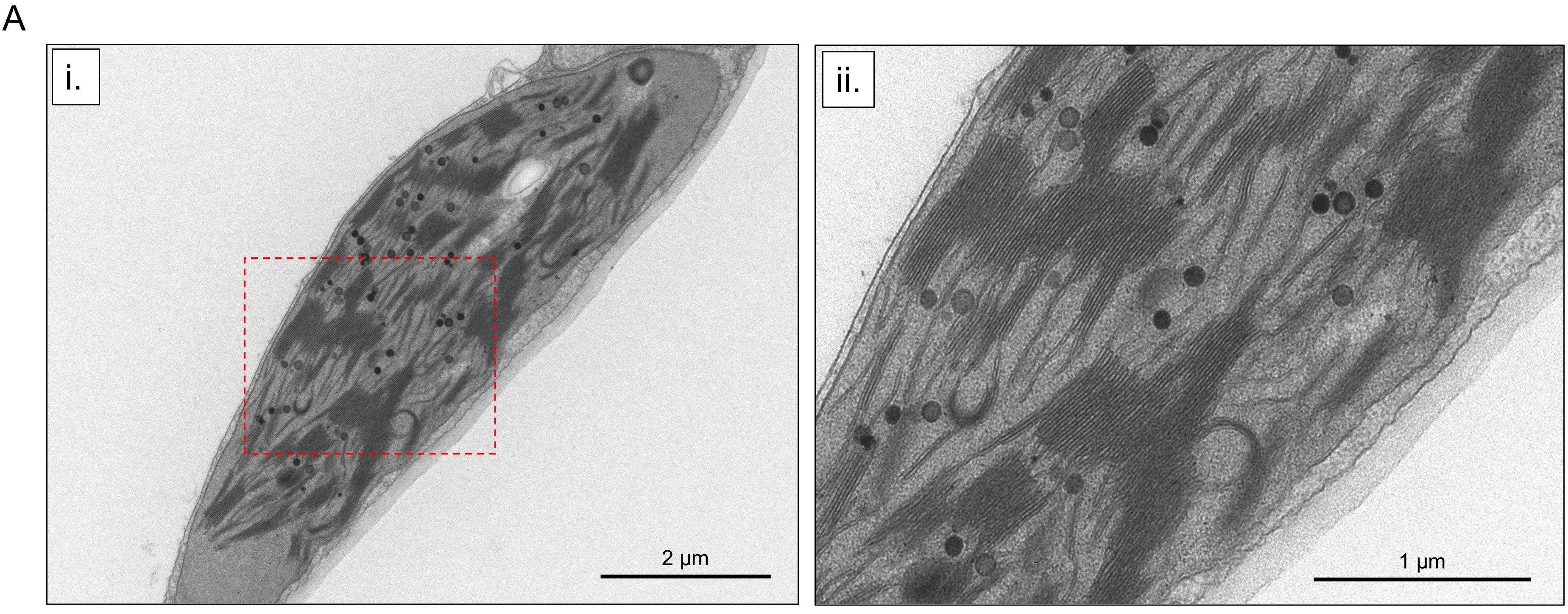

Supplement: msaf129_Supplementary_Data [file msaf129_supplementary_data.zip › Figure S6.JPG]

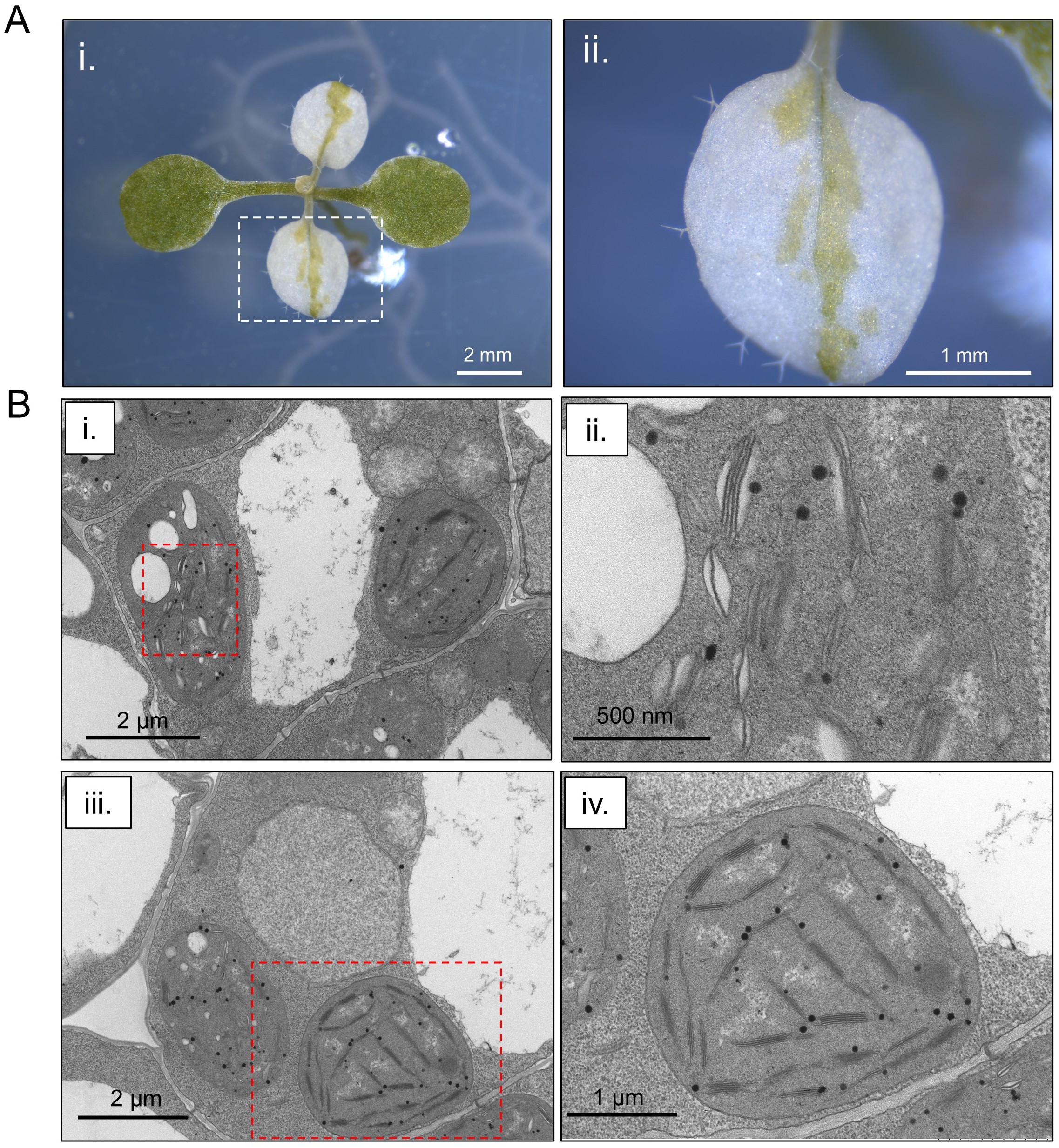

Supplement: msaf129_Supplementary_Data [file msaf129_supplementary_data.zip › Figure S7.JPG]

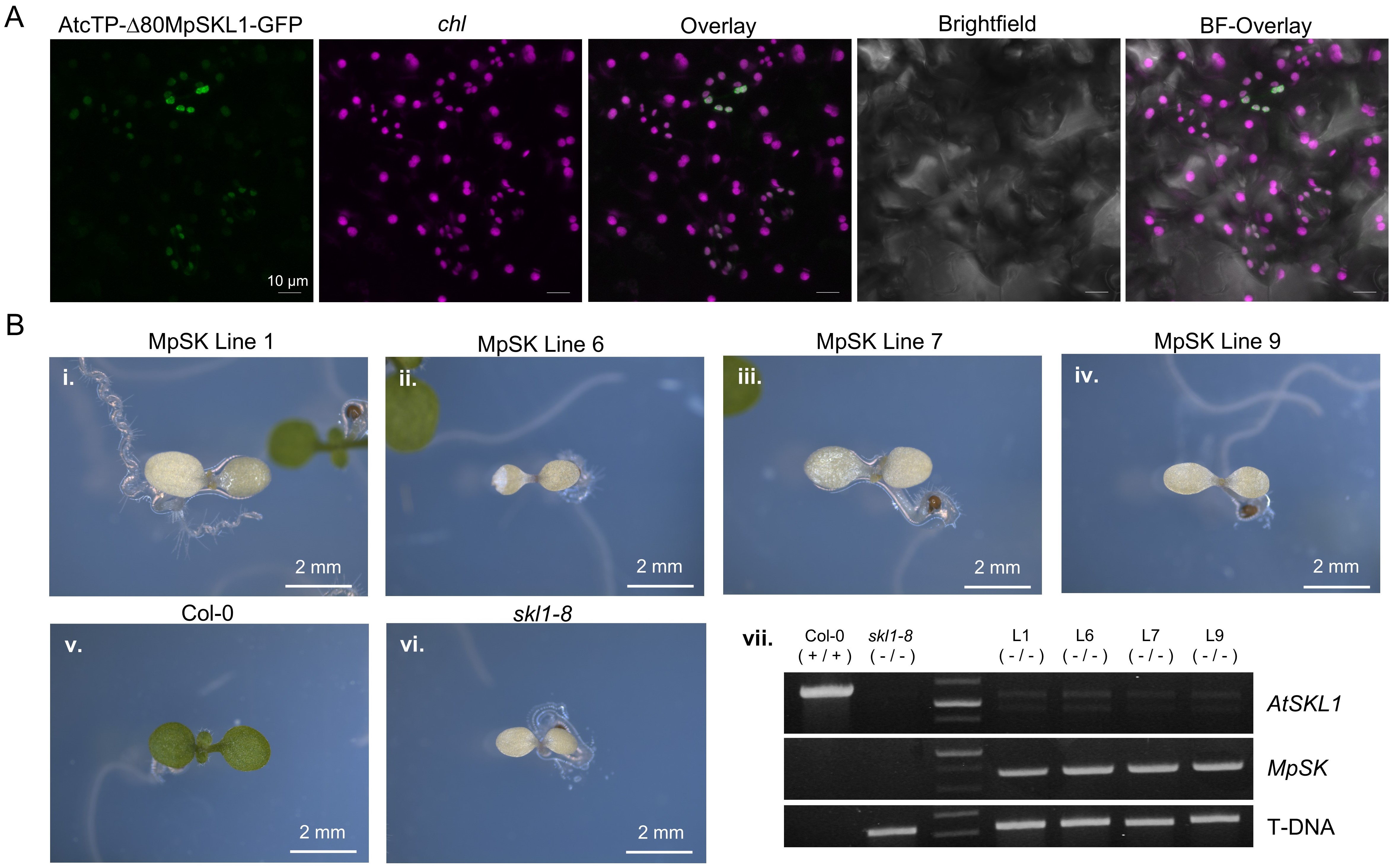

Supplement: msaf129_Supplementary_Data [file msaf129_supplementary_data.zip › Figure S8.JPG]

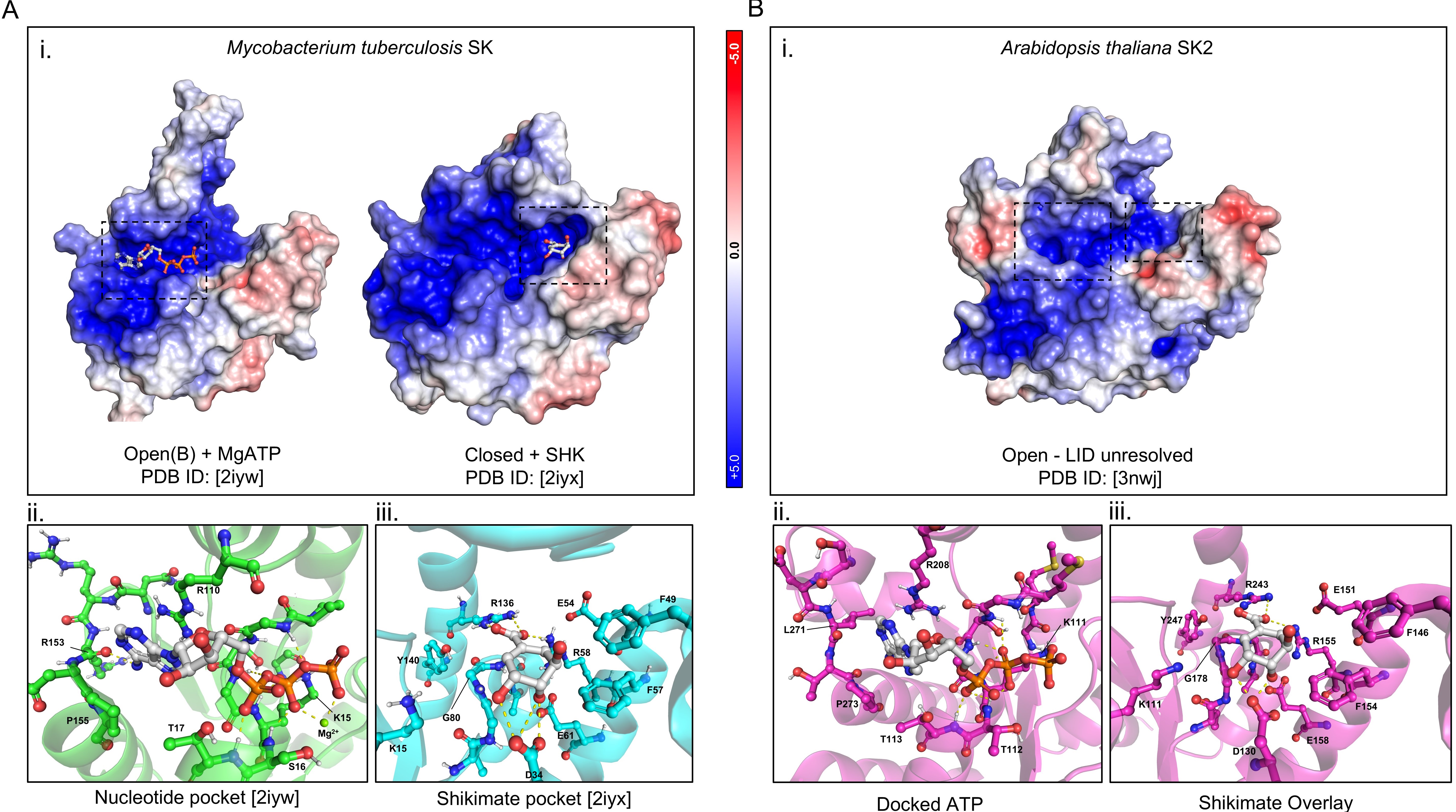

Supplement: msaf129_Supplementary_Data [file msaf129_supplementary_data.zip › Figure S9.JPG]
